# The policy implementation playbook: a cross-policy taxonomy of post-adoption tobacco industry tactics

**DOI:** 10.1186/s12992-026-01220-0

**Published:** 2026-06-12

**Authors:** Britta K. Matthes, Karen Evans-Reeves, Tom Gatehouse, Rosemary Hiscock, Iona Fitzpatrick, Anna B. Gilmore

**Affiliations:** Department for Health, University of Bath, Bath, UK

**Keywords:** Tobacco industry, Policy implementation, Tobacco control

## Abstract

**Background:**

Tobacco industry interference during policy development is well documented, yet evidence on how the industry responds after policies are adopted and enter into force remains fragmented across policy domains and jurisdictions. This study systematically examines post-adoption tobacco industry conduct across key tobacco control measures and develops a cross-policy taxonomy of post-adoption tactics. We conducted a scoping review and qualitative evidence synthesis of peer-reviewed and grey literature, searching six data bases and *Tobacco Control’s News Analysis* archive. Using inductive coding, we identified recurring forms of post-adoption industry activity and synthesised these into a conceptual taxonomy – the Policy Implementation Playbook (PIP).

**Results:**

We included 308 sources (210 peer-reviewed articles and 98 *News Analysis* items) spanning approximately 50 countries across all WHO regions, although documentation was concentrated in a limited number of settings. The PIP identifies five recurrent tactics. One – pre-emptive adaptation – occurs before a policy enters into force and includes stockpiling, transitional packaging, and early product or marketing adjustments. After a policy enters into force, the industry may disregard requirements, adopt token implementation that signals formal compliance while reducing practical impact, circumvent regulation through product-, design-, or channel-based tactics, or seek to influence implementation indirectly through retailers, hospitality actors, public authorities, and enforcers. Circumvention was the most frequently documented response across most policies, though its specific form varied across regulatory domains. Disregard and pre-emptive adaptation were also common, while token implementation was largely confined to health warning requirements. Smoke-free regulations more often elicited intermediary-focused strategies aimed at shaping interpretation, enforcement, and compliance.

**Conclusions:**

By conceptualising policy implementation as a contested political arena and synthesising recurrent tobacco industry tactics across policies, the PIP extends existing models of corporate political activity. The taxonomy provides a structured basis for anticipating post-adoption corporate conduct and strengthening regulatory design, implementation, and governance in tobacco control and the regulation of other unhealthy commodities.

**Clinical trial number:**

Not applicable.

**Supplementary Information:**

The online version contains supplementary material available at 10.1186/s12992-026-01220-0.

## Background

Tobacco control policies have been adopted across a growing number of countries since the turn of the century, driven in large part by the World Health Organization Framework Convention on Tobacco Control (WHO FCTC), which entered into force in 2005 [1]. For example, by 2024, 107 countries had achieved the highest levels of WHO FCTC implementation – defined as the translation of the treaty into national or local policy – in at least two policy areas [2].

The tobacco industry remains a principal barrier to policy progress [3]. Research shows that transnational tobacco companies act to prevent, weaken, or delay regulation that threatens their profits, particularly in the early stages of the policy cycle [4–6], where opposition is perhaps most visible. Common corporate political activities include lobbying, mobilising third parties, creating doubt, disseminating industry-favourable information, and deploying legal threats and challenges [7–9]. Frameworks such as the Policy Dystopia Model (PDM) [4, 10] illustrate how discursive and action-based strategies are used to resist and reshape policy proposals.

As tobacco control policies become more widespread and comprehensive, it is increasingly important to understand what happens after policy adoption. Evidence is clear that written policies can differ substantially from policies in practice, with studies indicating that key measures such as advertising bans and labelling requirements are often not fully enforced, particularly in lower-income settings [11–14]. Weak or uneven implementation can sustain commercial advantage and may reinforce industry claims of “anticipated failure” used to oppose regulation [8, 15].

In line with this, evidence suggests that tobacco industry interference does not cease once policies are in place. Studies document non-compliance [16–18], exploitation of loopholes [13, 19–21], and adaptive practices that preserve misleading connotations and can dilute policy impact [22–25]. However, this evidence remains fragmented across policy areas and jurisdictions, limiting the ability to identify recurring patterns or compare industry responses across different contexts. A notable exception is a systematic review of tobacco industry responses to excise tax policies, which mapped sophisticated pricing strategies used globally to undermine policy effectiveness [26]. While the PDM captures reputation and information management strategies that shape the policy environment across the policy cycle [4, 10], it does not systematically categorise the range of industry activities occurring once a policy has entered into force.

The present study addresses this gap by systematically examining and synthesising tobacco industry conduct after policy adoption across key tobacco control domains: tobacco advertising, promotion and sponsorship (TAPS), packaging and labelling, product-related regulation, and smoke-free policies (Articles 8–11 and 13 of the WHO FCTC). Packaging and labelling include misleading descriptor bans, health warning requirements, standardised packaging (also referred to as plain packaging), and minimum pack size regulations, while product-related regulation encompasses flavour restrictions, product bans, and ingredient disclosure requirements. These policy areas are particularly relevant because similar regulatory approaches are emerging for alcohol, ultra-processed foods, and other unhealthy commodities [27–33] and are included in the WHO’s “best buys” for non-communicable disease prevention, updated in 2024 [34].

A systematic understanding of industry tactics during implementation can help anticipate and pre-empt interference by identifying common adaptation strategies and regulatory vulnerabilities. By mapping and synthesising post-adoption industry activity across a range of tobacco control policies, this study aims to support more resilient and effective policy design, implementation, and enforcement. Given the similarities of pre-adoption corporate political activities across industries [15, 35], the findings may also offer insights relevant beyond tobacco.

## Methods

We conducted a scoping review and qualitative evidence synthesis of peer-reviewed and grey literature to develop an inductive taxonomy of post-adoption tobacco industry tactics, which we call the Policy Implementation Playbook (PIP). The review followed the *Preferred Reporting Items for Systematic Reviews and Meta-Analyses Extension for Scoping Reviews (PRISMA-ScR)* checklist.

### Peer-reviewed articles: identification and selection

#### Search strategy

We searched six databases *– Embase, International Bibliography of the Social Sciences (IBSS), JSTOR, PubMed, Scopus, and Web of Science –* covering health, social science, and interdisciplinary fields, using combined key terms related to the tobacco industry, policy, and implementation:

(“tobacco industry” OR “tobacco compan*”) AND (“Framework Convention on Tobacco Control”) AND (“law” OR “policy” OR “regulation” OR “act”) AND (“implement*” OR “enforc*” OR “compl*”).

This search string was developed through pilot searches. The term “Framework Convention on Tobacco Control” was included to improve precision while keeping results manageable and retaining global relevance.

Searches were conducted in December 2023 and updated in March 2025 and included all publication dates. English, German, or Spanish sources were eligible, reflecting the language proficiency of the research team.

#### Study selection

All references were imported into Covidence (*Veritas Health Innovation*) for screening and removal of duplicates. The lead researcher screened titles and abstracts to remove residual duplicates and exclude ineligible records, after which full texts were retrieved.

Following two pilot rounds, two reviewers independently screened the full texts against the eligibility criteria (Table 1) and recorded key reasons for exclusion. All discrepancies were resolved through discussion, with substantial agreement across reviewers.

**Table 1:** Eligibility criteria

Eligibility criteria	Examples of non-inclusions
To be included, a source had to report on one or more specific instances of tobacco industry activity that: • occurred in response to an adopted TAPS, packaging and labelling, product, or smoke-free policy; • could plausibly affect policy effectiveness; and • did not aim at repealing, delaying, or weakening a policy through formal policy reform.	- generic descriptions of industry activity (e.g. generic points that “the industry circumvents TAPS policies”)
	- activities attributed solely to other actors (e.g. non-compliant retailers) without an explicit or reasonably inferable industry link
	- activities with no policy trigger and those with other policy triggers (e.g. undermining tax policy)
	- activities aiming at repealing, delaying, or weakening a policy through formal reform processes, including those captured by the Policy Dystopia Model [4] (e.g. legal action, lobbying, or industry-generated evidence intended to facilitate such reform)

### News analysis: identification and selection

We also reviewed all available peer-edited *BMJ Tobacco Control News Analysis* sections published between 1992 and 2024, which provide documentation of tobacco industry activity that is often not captured in the academic literature.

We retrieved 167 complete and four incomplete *News Analysis *sections; one complete section from 1997 was missing. Missing pages could not be recovered despite contacting the publisher.

Following a pilot phase (five issues reviewed independently by two reviewers), the remaining issues were divided for screening against the eligibility criteria (see Table 1). Regular meetings and cross-checking ensured consistency.

#### Additional searches

To minimise the risk of missing relevant articles, including those not explicitly referencing the WHO FCTC, three complementary approaches were used to locate further evidence. First, backward searches were conducted by screening reference lists of all included articles and *News Analysis* items. Second, forward citation searches were conducted by identifying articles that cited included sources, using Google Scholar’s ‘cited by’ function. Third, targeted Google Scholar searches were undertaken in March 2025 using frequently occurring terms observed during data familiarisation (e.g. “circumvent”, “sidestep”, “subvert”), combined with “tobacco control”. Newly retrieved sources were screened using the eligibility criteria (Table 1).

#### Data extraction

A structured proforma was developed in Excel to extract key details, including the industry activity described, relevant policy and geography. Details from all included sources were added to the proforma.

### Data charting and analysis

The approach to data charting and analysis was informed by existing frameworks on corporate political activity [4, 8, 9, 15], but aimed to extend these to the post-adoption stage. Analysis followed an inductive and iterative logic.

In a series of author meetings, an initial set of descriptive categories was developed to summarise recurring patterns in the data and organised into a draft taxonomy. Two coders tested the draft taxonomy. Once a shared understanding was reached, they mapped the remaining sources. Where a single instance involved multiple tactics, it was mapped to the primary tactic most directly shaping policy implementation.

To ensure consistency, the two coders met regularly and a subset of data was double coded in each policy area (10%-50%, depending on subset size). Categories and definitions were iteratively refined until stable, resulting in a coherent taxonomy presented in the Results.

For the final analysis, policy areas were subdivided by policy type where the literature permitted meaningful differentiation. Packaging and labelling policies were categorised into misleading descriptors, health warnings, standardised packaging, and pack size, while product-related regulation was grouped into flavour restrictions, product bans, and ingredient disclosure. Such subdivision was not feasible for TAPS policies because many sources did not specify whether policies covered advertising, promotion and/or sponsorship, and terms such as “marketing”, “advertising” and “promotion” were frequently used interchangeably. For smoke-free policies, the dataset was comparatively small and the policy measures described were largely homogeneous, making further categorisation unnecessary.

## Results

**Fig. 1:**
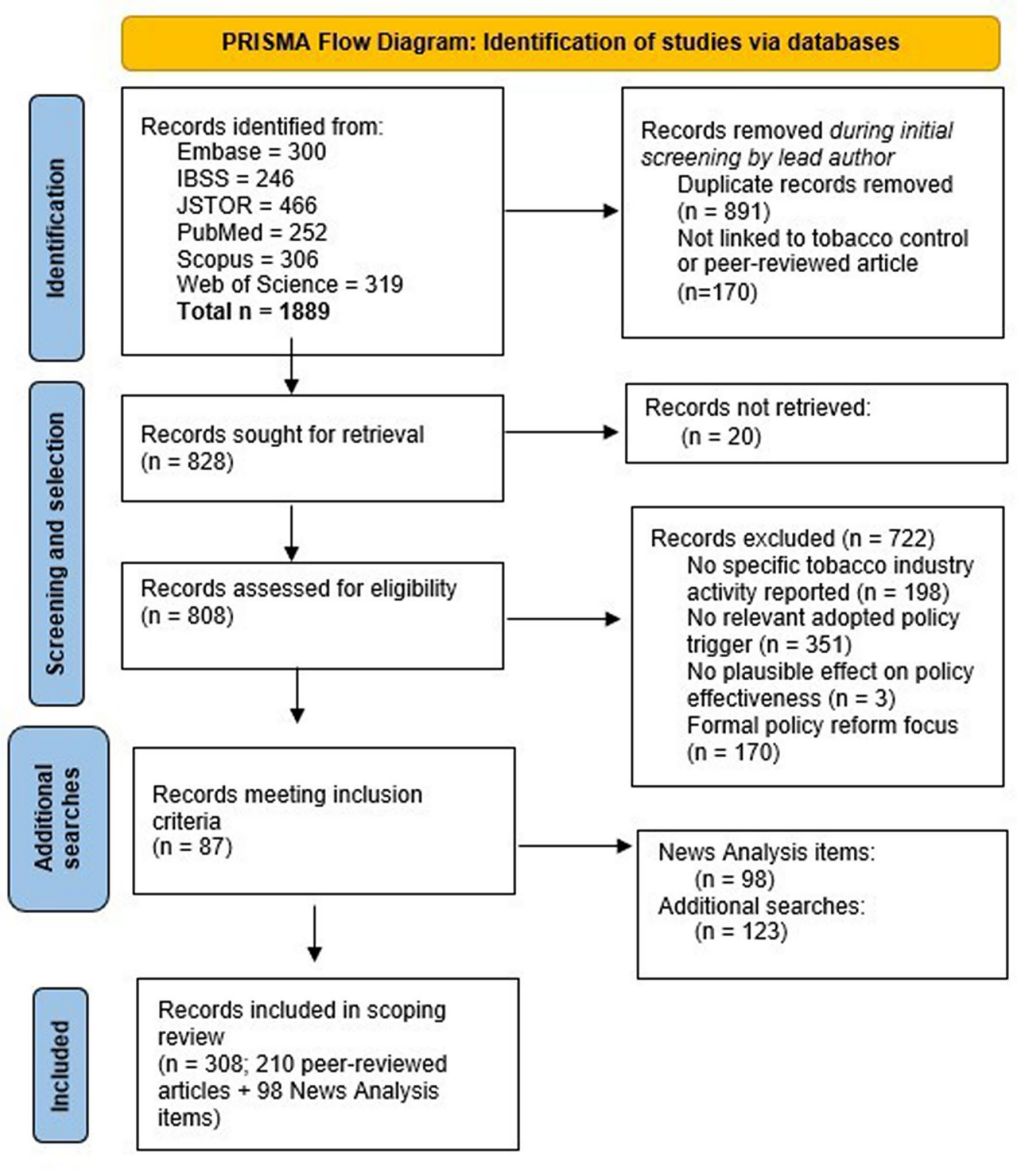
PRISMA flow diagram (based on [36])

Figure 1 presents the PRISMA flow diagram [36] for this review.

### Overview of the sample

In total, 308 sources (210 peer-reviewed articles and 98 News Analysis items) were included in the study (see Supplementary material, Table 1). These sources documented tobacco industry activity across approximately 50 countries and spanned all WHO regions, although coverage was uneven. The evidence base was concentrated in a small number of countries, most commonly in the United States of America (USA), Australia, India, the United Kingdom (UK), Canada, and Thailand. While several sources specified one or more tobacco companies – most commonly transnational tobacco companies and their subsidiaries – others referred more broadly to the “tobacco industry”.

Most sources described tobacco industry responses to TAPS policies (211/308, 69%), followed by packaging and labelling (77/308, 25%), product-related (34/308, 11%), and smoke-free regulations (9/308, 3%), with some sources addressing more than one policy area.

**Fig. 2:**
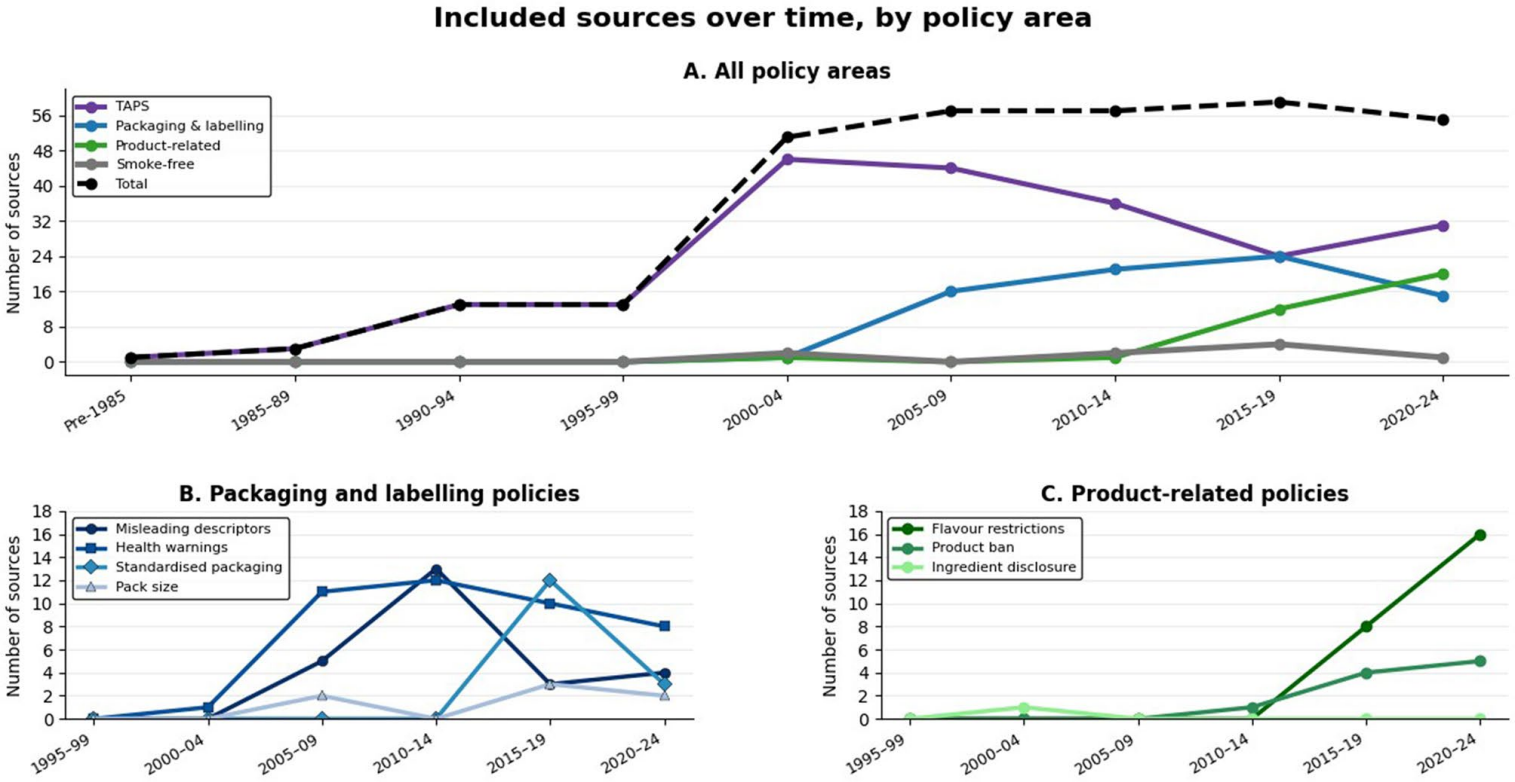
Included sources over time, by policy area. Legend: Panel **A** shows the number of included sources across policy areas, grouped into five-year publication periods (with pre-1985 years combined). Panels **B** and **C** show the number of included sources by specific policy types within packaging and labelling policies and product-related policies, respectively. The dashed line in Panel **A** indicates the overall number of included sources across all policy areas in each publication period. Some sources are included in more than one policy area; counts across policy areas should therefore not be summed

Included sources were published between 1971 and 2024. The number of included sources increased sharply between 1995-1999 and 2000–2004 and remained high thereafter (Fig. 2). Sources published in earlier periods were dominated by TAPS policies. From the mid-2000s onwards, publications addressing packaging and labelling increased steadily, while sources focusing on product-related regulation emerged primarily after 2015–2019. Smoke-free regulations accounted for a small proportion of sources across all time periods.

### Introducing the Policy Implementation Playbook (PIP)

The Policy Implementation Playbook (PIP) synthesises post-adoption tobacco industry activities aimed at undermining policy effectiveness into five tactics (Table 2).

**Table 2:** The Policy Implementation Playbook

Timing	Tactic	Sub-tactic	Definition	Illustrative examples
Prior to entry into force	**1. Pre-emptive adaptation**		Actions taken before or immediately around a policy entering into force to position brands or products in ways that reduce the impact of the incoming regulation.	Stockpiling non-compliant products; introducing “transition” packaging or accessorie
After entry into force	**2. Disregarding policy**		The tobacco industry ignores a legal requirement, or treats it as non-binding, despite regulatory clarity.	Continued sale or promotion of products after a ban has entered into force
	**3. Token implementation**		Superficial or minimal actions that signal formal adherence while preserving the practical effect of the prohibited activity.	Using removable or low-visibility health warnings
	**4. Circumventing policy** (Industry adaptation that undermines policy intent by **exploiting or creating regulatory ambiguities**, regardless of subsequent legal clarification or rulings. It is subdivided analytically by **what is modified (4.1.)**, **how meaning is conveyed to consumers (4.2.)**, and **where activity shifts (4.3.)**)	4.1. Product-based	Changes to tobacco products, product categories, or ancillary items that enable restricted attributes, functions, sensory effects, *or* market positioning to be preserved or reintroduced.	Selling modified products or components separately so they fall outside legal definitions
		4.2. Design-based	The use of alternative means (e.g. design elements, imagery, colours, shapes, layouts or symbolic references) to communicate restricted attributes or brand/product recognition to consumers.	Replacing banned descriptors with colours, symbols, or layout changes that convey similar meanings
		4.3. Channel-based	Shifting or maintaining product- or brand- related promotion by relocating promotional or engagement activity into alternative environments, platforms, or settings that remain weakly regulated, exempted, or ambiguously covered.	Shifting promotion into retail displays, digital platforms, sponsorship, or experiential marketing
	**5. Influence through intermediaries** (Industry actions aimed at **shaping how regulation is interpreted, implemented, and enforced by other actors**)	5.1. Retailers	Shaping implementation through retailers, distributors, and similar actors.	Offering financial incentives or guidance shaping product placement, displays, or information provided to consumers
		5.2. Hospitality actors	Encouraging hospitality owners or staff to undermine policy effectiveness.	Encouraging bars or restaurants to permit prohibited practices
		5.3. Public authorities and enforcers	Influencing public authorities or enforcers to interpret and enforce the policy in an industry-favourable way.	Promoting favourable interpretations of rules or discouraging active enforcement

Legend: The table presents a taxonomy of tobacco industry tactics observed after policy adoption, organised by timing, tactic, and sub-tactic. Definitions describe underlying mechanisms, while illustrative examples indicate how tactics may manifest in practice. Examples are non-exhaustive

The PIP distinguishes between activities occurring before and after a policy enters into force. Pre-emptive adaptation captures activities undertaken prior to entry into force, while the remaining tactics occur thereafter. These include disregarding policy,token implementation, circumventing policy – further classified into *product-based, design-based*, and *channel-based* forms – and efforts to influence implementation through intermediaries, including retailers, hospitality actors, and public authorities and enforcement staff.

### The Policy Implementation Playbook (PIP) across policy domains

Across most policy areas, circumvention was the most frequently documented tobacco industry response, although the dominant subtype varied (Table 3). *Channel-based circumvention* predominated for TAPS, while *design-based circumvention* was most common for packaging and labelling measures (except for pack size regulation). *Product-based circumvention* was equally common for standardised packaging and was the most frequently documented tactic in response to flavour restrictions and product bans.

**Table 3:** The Policy Implementation Playbook (PIP) across policies

	TAPS policies	Packaging and Labelling policies					Product-related policies				Smoke-free policies
	Total	Total	Misleading descriptor bans	Health warnings	Standardised packaging	Pack size restrictions	Total	Flavour restrictions	Product bans	Ingredient disclosure requirements	Total
Pre-emptive adaptation	9 (4%)	18 (23%)	6 (24%)	4 (10%)	8 (53%)	2 (29%)	10 (29%)	9 (36%)		1 (100%)	
Disregard	53 (25%)	20 (26%)	7 (28%)	16 (38%)		2 (29%)	8 (24%)	4 (17%)	4 (40%)		1 (11%)
Token implementation	3 (1%)	18 (23%)		16 (38%)		2 (29%)	2 (6%)	1 (0.5%)		1 (100%)	
Circumvention	169 (80%)	45 (58%)	16 (64%)	19 (45%)	12 (80%)		25 (74%)	18 (75%)	6 (60%)	1 (100%)	3 (33%)
*-Product-based*	*9 (4%)*	*14 (18%)*	*3 (12%)*	*3 (7%)*	*8 (53%)*		*24 (71%)*	*17 (71%)*	*6 (60%)*	*1 (100%)*	*2 (22%)*
*-Design-based*	*29 (14%)*	*35 (45%)*	*15 (60%)*	*14 (33%)*	*8 (53%)*		*6 (18%)*	*6 (25%)*			
*-Channel-based*	*138 (65%)*	*6 (8%)*	*1 (4%)*	*3 (7%)*	*2 (13%)*		*3 (9%)*	*3 (13%)*			*1 (11%)*
Influence through intermediaries	17 (8%)	8 (10%)	1 (4%)	2 (5%)	4 (27%)	1 (14%)	3 (9%)	2 (8%)	1 (10%)		5 (56%)
*-Retailers*	*15 (7%)*	*8 (10%)*	*1 (4%)*	*2 (5%)*	*4 (27%)*	*1 (14%)*	*3 (9%)*	*2 (8%)*	*1 (10%)*		
*-Hospitality actors*	*1 (,%)*										*2 (22%)*
*-Authorities/enforcers*	*1 (,%)*										*3 (33%)*
Total number of sources	211	77	25	42	15	7	34	24	10	1	9

Legend. The table presents the number of included sources documenting tobacco industry responses to different policies, organised by the PIP tactics and sub-tactics. Percentages indicate the proportion of sources reporting a given tactic. Some sources are included under more than one policy domain and/or tactic; counts across rows or policy domains should therefore not be summed

Disregarding policy was also frequently documented across most policy types. In contrast, token implementation was concentrated primarily in relation to health warnings on packs and in advertising materials, reflecting superficial adherence without substantive change. Pre-emptive adaptation and efforts to influence implementation through intermediaries were observed across multiple policy areas. The latter was proportionally more prominent in smoke-free policies, although the small number of smoke-free sources limits direct comparison.

### The Policy Implementation Playbook (PIP) by policy area

#### Tobacco advertising, promotion and sponsorship (TAPS) policies

Pre-emptive adaptation involved actions undertaken before or immediately around the introduction of TAPS restrictions. Activities included last-minute intensification of marketing through advertising “blitzes” and high-impact visual displays (e.g. [37–39]). Companies also sought to embed brand presence within the retail environment ahead of restrictions, for example through shopfront redesigns using brand colours, more prominent or diversified display units, and the pre-installation of unit covers (e.g. [40–42]). Other activities included testing indirect advertising and trademark diversification strategies, consolidating brand portfolios, and collecting consumer data through nightlife promotions prior to their prohibition (e.g. [43–45]).

Disregarding TAPS policies was repeatedly documented and included industry continuation of advertising and point-of-sale (PoS) marketing; (e.g. [46–48]) sponsorship of sports, music, and youth-oriented events; (e.g. [49–51]) and the distribution of free tobacco products, branded merchandise, and promotional incentives (e.g. [52–54]). In several instances, these activities prompted civil society intervention and regulatory action, including investigations, administrative fines, and court rulings.

Token implementation was rarely documented and was primarily linked to health warnings (HWs) in advertisements in LMICs. In Pakistan, HWs were too small [55], in South Africa, newspaper advertisements displayed packs in ways that reduced the size of HWs [56], and in Nigeria, selective formatting minimised HW visibility in magazine advertisements [57].

Circumvention tactics dominated industry responses. *Channel-based circumvention* activities were the most frequently documented response to TAPS regulations, with marketing activity relocating into channels that remained partially permitted, weakly regulated, or difficult to enforce.

Retail and other commercial environments: PoS and surrounding retail space emerged as central sites of promotion across jurisdictions (e.g. [58–63]). Following regulations that substantially restricted non-retail tobacco advertising – including those deriving from the 1998 Master Settlement Agreement (MSA) in the USA – expanded cigarette displays; dense in-store and storefront advertising; and intensive PoS promotion in convenience stores, petrol stations, kiosks, and roadside stalls were observed (e.g. [64–66]). Across jurisdictions, companies used oversized or clustered PoS boards, brand-highlighted price boards, colour-coded and illuminated displays, and large three-dimensional installations such as power walls and gondola-end “spectaculars” (e.g. [63, 65, 67–69]). Other practices exploited definitional ambiguities, including the use of electronic screens and “commercial information” exemptions [70], “live stock” displays [40], and transparent cabinets [7].

Media channels: After the 1971 TV and radio advertising ban in the USA, tobacco companies intensified advertising in print media – particularly women’s and youth-oriented magazines – using larger formats, repeated placement, and highly visual imagery [71, 72]. After the MSA, companies concentrated advertising in publications with large absolute numbers of youth readers while remaining formally compliant with youth-readership thresholds [73, 74]. Similar practices were documented elsewhere, including indirect radio promotion in Sri Lanka [75]. Tobacco companies also used cross-border media spillover, including satellite television and foreign broadcasts (e.g. [24, 25, 45, 76]). More recently, marketing has shifted toward digital and social media platforms, including branded websites, influencer collaborations, email marketing, and interactive content (e.g. [77–81]).

Events and institutional platforms: After advertising and promotion restrictions were adopted, tobacco companies used elite and mass sporting sponsorship – particularly Formula 1, football, and cricket to secure repeated brand exposure via race coverage, stadium signage, and associated media content (e.g. [44, 82–85]). Companies also exploited sponsorship exemptions and cross-border broadcasting, and used indirect imagery [24, 25, 60, 86]. A related set of practices centred on music, nightlife, and youth-oriented lifestyle events, including concerts, club nights, fashion events, and bar-based promotions, often combined with sampling, discounts, and database-building (e.g. [49, 77, 78, 87–89]). In some LMICs, beauty pageants and youth party events were also used as promotional platforms [90, 91]. Cultural, educational, and institutional sponsorship – including film festivals, fashion weeks, art competitions, universities, and awards ceremonies – also served promotional purposes (e.g. [92–96]). Corporate social responsibility initiatives used foundations, charity programmes, and school-related activities to display corporate names and logos and generate favourable media coverage (e.g. [97–99]).

Direct engagement: Following Australia’s 1992 advertising ban, Philip Morris used affinity and relationship marketing platforms (*Waves and Wavesnet*) requiring consumer registration, enabling data collection and direct communication about tobacco-related events and promotion [77, 78]. Face-to-face engagement strategies also included actors posing as “tobacco experts” to distribute free samples in bars and restaurants [100], the deployment of brand “ambassadors” at entertainment venues [101], and the covert promotion of cigarettes within university settings through disguised “research” activities [102].

*Design-based circumvention* activities were less commonly reported and primarily involved the use of cigarette packs as substitute advertisements. Across multiple countries, companies used distinctive colours, imagery, shapes, limited editions, pack inserts and onserts (including wrappers and other promotional materials attached to or wrapped around packs, often promoting contests or digital engagement), altered opening mechanisms, and variant differentiation, using the pack as the advert (e.g. [43, 49, 87, 103–107]). In some settings, branding elements were printed directly onto cigarette sticks, exploiting them as residual promotional spaces [108].

Related practices involved indirect brand signalling without explicit advertising, relying on visual cues and brand recall. This included adaptations in Formula 1 sponsorship following advertising bans, where cigarette brand names were replaced with slogan-style substitutes and brand-linked colour schemes [109, 110], such as Philip Morris International’s (PMI) Mission Winnow campaign – a slogan-style campaign using Marlboro-linked colours and design elements [111] – as well as non-tobacco products and corporate branding arranged to visually evoke cigarette branding [60, 112–114].

*Product-based circumvention* activities were least frequently documented. In some cases, companies introduced substitute products not covered by regulation. For example, after the 1971 TV advertising ban in the USA, some companies introduced unregulated substitutes like little cigars – designed to mimic cigarettes in appearance and use [115]. More recently, companies have shifted marketing emphasis toward heated tobacco products (HTPs) not covered by comprehensive TAPS restrictions in many countries [116]. Companies also promoted ancillary non-tobacco products excluded from advertising bans, including rolling papers and filter tips [117].

Other adaptations exploited definitional and scope-related loopholes, including the separate sale of heated tobacco device components [118], the marketing of nicotine-containing products outside statutory definitions of tobacco [119], and the promotion of snus by asserting that it was not legally defined as a tobacco product [120]. Further adaptations involved changes to product formats and variants that enabled indirect brand promotion under advertising bans, including smaller or non-standard pack sizes [121, 122] and the use of menthol or “light” variants as extensions of existing brands [123].

The tobacco industry also sought to influence implementation through intermediaries, predominantly *retailers*. For example, companies provided financial and material incentives – including price discounts, rebates, gifts, free or exchangeable stock, loyalty schemes, and experiential rewards – in exchange for brand prominence, influence over product range and pricing, and compliance with company-defined merchandising practice (e.g. [41, 62, 65, 124, 125]). Companies also controlled retail environments by supplying gantries, cabinets, price lists, and customised hardware; advertising directly to retailers through trade publications; and undertaking in-store monitoring to ensure adherence to industry-specified display, pricing, and promotional requirements [20, 65, 126]. In a small number of cases, companies sought to shape retailer perceptions by providing reassurance or misinformation regarding regulatory requirements and enforcement [127, 128].

Beyond retailers, the industry provided financial incentives to *bar and nightclub owners* to host cigarette promotions [129]. In Hungary, tobacco companies influenced *enforcement authorities’* interpretation of advertising bans by promoting an expansive definition of PoS advertising [130]. This interpretation, supported by the Ministry of Economic Affairs, led the Consumer Protection Directorate to permit tobacco advertising visible from public space, contrary to the intent of the law, until it was overturned by court rulings.

#### Packaging and labelling policies

##### Misleading descriptor (MD) bans

Pre-emptive adaptation included tobacco companies registering trademarks incorporating MDs into brand names [131], stockpiling non-compliant products [132], and using transition packs – via cellophane wrappers, pack inserts and onserts, and retailer communication – to signal forthcoming changes [133, 134]. New brands replaced banned MDs with colour names while leaving other packaging unchanged to ensure continuity [135, 136].

Disregarding policy, products with MDs were found for sale in stores in Asia, Eastern Europe, Oceania and South America [12, 13, 135, 137, 138], and in duty free shops in Mauritius [132]. In Southeast Asia, PMI continued to register brand names with MDs [139].

Circumvention tactics were frequently documented, particularly d*esign-based* tactics that replaced banned descriptors with colour and other evocative terms. For example, ‘smooth’, ‘subtle’ and ‘superfine’ were used to replace ‘lights’, and colours such as blue and gold were used for the same purpose [22, 97, 131, 133, 140–147]. Some brand names incorporated filter innovations too, e.g. ‘Triple Filter charcoal’ [131]. Promotional phrases highlighting innovations were added to the packaging and wrapping [7, 131, 148, 149], information on the packs was reorganised to provide greater white space and packaging thickness was used to suggest premium quality [133, 145]. Cellophane wrappers also served to colour packs and link to promotional websites [149].

*Product-based circumvention* included cigarette filter innovations to suggest reduced harm. In the USA, tobacco companies increased the number of brand variants with filter ventilation holes [150] and made filter ventilation correspond with colour names which have health-related connotations [140]. Similar tactics were observed in Malaysia with the introduction of capsule filters [131].

*Channel-based circumvention* included the use of interactive online promotional content where users could play games, take part in competitions and receive coupons for tobacco products (RJ Reynolds and Camel; PMI and Marlboro) [149].

Finally, tobacco companies sought to influence implementation through intermediaries - using *retailers* in Mexico to explain changes to customers and avoid confusion [133].

##### Health warning (HW) requirements

Pre-emptive adaptation included stockpiling old products [121], the release of special edition products in reusable HW-free tins [151], and giveaways of metallic covers or long-lasting plastic covers designed to obscure graphic health warnings (GHWs) [152, 153].

Disregarding policy, smoked and smokeless tobacco products without HWs or with outdated HWs were sold in multiple countries [7, 12, 13, 17, 131, 132, 137, 154–160]. In Indonesia, tobacco companies failed to submit required HW images by the implementation deadline [161]. More recently, PMI marketed HTP devices as non-tobacco products without GHWs in some jurisdictions, including Lithuania, contrary to national regulations [118].

Token implementation was widely documented and involved superficial compliance that reduced the visibility or salience of mandated warnings. This included the use of languages not understood by most locals, warnings below the required minimum size, poor-quality or manipulated images (e.g. blurred, faded, tinted, or visually minimised), failures to rotate warnings, and incorrect placement on packs, collectively preserving brand appeal [12, 137, 154, 159, 162–166]. In Kenya, HWs were applied as removable stickers [5] and in Indonesia, the top five brands lacked the mandated statement that there is no safe level of tobacco consumption [157]. In some cases more packs appeared to have the least impactful warnings, suggesting unequal printing [167, 168].

Circumvention tactics were frequently documented, particularly *design-based strategies* involving pack modifications that reduced the visibility of GHWs. These included book-style packs (Chile), hard covers and double packs (Mexico), lipstick packs and colourful sleeves (Malaysia), flip top and split packs that divided or removed warnings (Canada and Australia). Companies also used colours, textures, seals, wrappers, pack insides and promotional phrases to distract from or obscure warnings [7, 121, 148, 152, 169–175]. In addition, tobacco companies sold branded metal tins without GHWs (Indonesia) or with peelable HWs (Australia) [157, 176].

*Product-based circumvention* included the proliferation of cigarette-like cigars exempt from HW requirements in the USA [115], and as aforementioned, the introduction of HTPs such as IQOS, with devices sold separately from tobacco sticks to circumvent regulations applying to tobacco products [118, 177].

*Channel-based circumvention* involved obscuring HWs through advertising and retail display practices, including images of open packs in advertisements (South Korea), deliberate power-wall arrangements (Australia), and PoS displays that diverted attention (Philippines) [178–180].

Finally, the industry sought to influence implementation through intermediaries by misleading *retailers* in Southeast Asia about enforcement dates and the permissibility of selling old stock, delaying the practical impact of HW regulations [7, 157].

##### Standardised packaging

Pre-emptive activities were extensive. In several countries long sell-off periods for branded packs meant these were on sale right up until the implementation deadline. Buy-back schemes enabled retailers to stockpile branded products and popular brands appeared in standardised packaging last. Companies also introduced reusable tins and modified packs to communicate brand continuity and forthcoming changes, including variant name changes and product innovations [22, 181–187].

Disregard of policy and token implementation were not documented. However, circumvention tactics were widely documented, with product- and design-based strategies most frequently reported.

*Product-based circumvention* included the introduction of cigarette-like cigarillos sold in branded packs, exploiting their exemption from standardised packaging legislation in the UK [19]. Tobacco companies also introduced filter innovations designed to signal quality or differentiated experiences, including capsule filters, recessed filters, and extended cigarette lengths [22, 182, 188, 189], with similar practices observed in Australia and Singapore [23, 190]. Other product innovations included mixing tobacco with mint leaves [183].

*Design-based circumvention* preserved brand differentiation and reduced the salience of standardised packaging through pack modifications. Companies replaced former pack colours with colour-coded variant names and experience-based descriptors, highlighted filter innovations on packs, and altered typography to increase the prominence of variant names [23, 182–184, 190–192]. Additional strategies included changes to pack shape and structure – such as slim, elongated, soft, or bevelled packs – and modifications that concealed or disrupted HWs, including foil packs without HWs, packs that concealed the HW when opened, and cellophane stickers [23, 182–184, 189, 190]. In some cases, packs technically complied with size requirements while failing to meet HW specifications, such as slim packs in the UK [182], or were suspected of incorporating prohibited sensory features, such as scented tear tape in Singapore [23].

*Channel-based circumvention* maintained brand communication through retail practices, including gantry stickers and price boards that displayed full brand lists despite restrictions on pack branding [19, 181].

Finally, tobacco companies sought to influence implementation through intermediaries by strengthening relationships with *retailers*, offering incentives for prominent product placement, maintaining fully stocked gantries, and providing higher profit margins for new cigarillo products, thereby aiming to maximise sales under standardised packaging regimes [19, 22, 181, 188].

##### Minimum pack size requirements

Pre-emptive adaptation included tobacco companies launching small tins designed to hold ten cigarettes and smaller roll-your-own tins in anticipation of minimum pack size restrictions in the UK [22, 187].

Disregarding policy, cigarettes were found for sale in packs smaller than permitted, including 10-packs in Guatemala and Myanmar [154, 193].

Token implementation involved practices that formally met numerical requirements while preserving affordability and accessibility. In the Philippines, bundles of four small packs containing five cigarettes each were sold together, often displayed in strips similar to confectionery products [194]. Although the bundles contained a total of 20 cigarettes, advertising highlighted the price of a single five-cigarette pack, and the sale of individual small packs was witnessed. In Australia, “twin wallet” packs (also referred to as “kiddie packs”) could be separated into two smaller packs of 13 and 7 cigarettes, facilitating shared purchases and increased affordability among young people [172].

Finally, tobacco companies sought to influence implementation through intermediaries by providing branded kiosks and umbrellas to *informal traders* selling single sticks in African countries [54].

#### Product-related policies

##### Flavour restrictions

Flavour restrictions, which in some jurisdictions refer to bans on specific ingredients and in others to prohibitions on characterising flavours, were met with multiple tobacco industry responses.

Pre-emptive adaptation was common and reflected patterns also observed in later circumvention efforts. Activities included registering or introducing new products and accessories, such as menthol cigarillos and clove cigars [21, 195], accessories such as flavour cards [196–198], and new capsule brands [20, 22, 199]. In Canada, stickers were placed on cigarette wrappers directing consumers to non-menthol alternatives [199], while in Denmark menthol pipe tobacco, electronic cigarettes (e-cigarettes), and HTPs were marketed as substitutes for banned products [21].

Disregarding policy, products marketed as “non-menthol” but containing high levels of menthol were introduced following the EU/UK characterising flavour ban [200, 201]. In the UK, brands marketed as “fresh” were reported to taste of menthol [196], and in Sri Lanka, flavoured capsule cigarettes were sold after the flavoured cigarette ban [202], prompting government investigations.

Following the EU characterising flavour ban and concerns over non-compliance in Denmark, Japan Tobacco International (JTI) stated that it continued to use menthol in compliance with the law (i.e., no prominent smell or taste of menthol) (token implementation) [21].

Circumvention tactics were widespread after flavour restrictions, with *product-based circumvention* dominating. Companies relabelled and adapted products such as flavoured little cigars and cigarillos to resemble cigarettes, while keeping them outside the scope of flavour bans [19, 20, 196, 203–205]. In the UK, PMI marketed its flagship HTP as “the closest alternative to menthol cigarettes”, supported by promotional menthol kits and consumer trials [20, 196].

Other product-based strategies focused on preserving or reproducing flavour effects within the scope of regulation. These included the introduction of flavour capsules and filter innovations; menthol, mint or fruit filter tips; recessed or crush filters; and other mechanisms designed to release or mimic flavour during use [18, 20–22, 188, 196, 206–209]. In Denmark, menthol aroma was added to pack lids, which was later ruled non-compliant [21].

Companies also reformulated products to reproduce cooling sensations, employing synthetic cooling agents, altering filter ventilation or tobacco weight, and introducing “cooling” or “fresh” non-menthol variants offering similar sensory experiences [21, 200, 210]. Reformulation was also used to evade legal definitions. In the USA, “tobacco-free nicotine” products made with synthetic or highly purified nicotine were introduced to avoid restrictions on electronic nicotine delivery systems (ENDS), while in South Korea manufacturers used nicotine extracted from tobacco stems or roots to circumvent the legal definition of tobacco as products derived from the tobacco leaf [18].

Flavour was additionally reintroduced at the point of use through sensory accessories, including flavour cards, drops, sprays, inserts, menthol-flavoured stones, roll-your-own tubes, and flavour caps fitted over cigarette sticks [18, 20, 21, 198, 208].

*Design-based circumvention* included colour descriptors in product names (e.g. “blue”), menthol-associated packaging cues (green or blue colour schemes, “smooth” descriptors), and slogans such as “smooth taste redesigned” or “the non-menthol for menthol smokers” on wrappers and packs, which communicated continuity [207, 211, 212].

*Channel-based circumvention* targeted retail and marketing environments to promote replacement products. In California, gantries and billboards advertised “non-menthol” lines closely mimicking menthol branding, while in-store price promotions incentivised their sale [211]. Direct mail and website marketing also targeted menthol consumers with new variant lines [208, 210].

Finally, tobacco companies sought to influence implementation through intermediaries by marketing substitute products and new brands directly to *retailers*, including through retail magazines in the UK [20, 188].

##### Product bans

Pre-emptive adaptation and token implementation were not documented.

Disregarding policy, despite the ban on gutka (a commercially produced form of smokeless tobacco) in India and the ENDS ban in Brazil, banned products remained widely available [18, 213]. In Thailand, tobacco companies continued to apply for trademarks for HTPs and ENDS despite bans [139]. In India, following the ban on newer products, PMI promoted HTPs as ‘safer alternatives’ and offered research grants on e-cigarettes and harm reduction [214].

Circumvention tactics all referred to *product-based circumvention* of the Indian gutka ban. This involved companies selling tobacco and non-tobacco components in separate packs, enabling consumers to combine them post purchase; often, the tobacco component was offered as a free add-on [13, 215–217]. Companies also intensified marketing and free distribution of alternative products, such as mawa - another smokeless tobacco product - that were not covered by the ban [218]. In addition, a mouth freshener – featuring similar colour schemes, logos, and fonts as the companies’ earlier gutka pouch – was launched, and the mouth freshener brand was then used to co-present the broadcast of sports events and co-sponsor a major film event [51].

Finally, companies sought to influence the implementation of the gutka ban by marketing alternative products through intermediaries (retailers) [218].

##### Ingredient disclosure

In Thailand, the tobacco industry responded to ingredient disclosure requirements through multiple tactics [219]. Pre-emptive adaptation reportedly involved efforts to ensure the continued availability of illicit tobacco products prior to implementation, maintaining consumer access outside the regulated market. Token implementation occurred through the provision of generalised ingredient lists that obscured full product composition. *Product-based* circumvention was also documented, with products reformulated for the Thai market to avoid full disclosure and protect proprietary recipes.

#### Smoke-free policies

Pre-emptive adaptation and token implementation were not documented.

Disregarding policy, in Brazil, despite national smoke-free regulations for indoor public spaces, British American Tobacco (BAT) installed a branded “smoking point” inside an airport [220]. The structure combined a designated smoking facility with company logo display and consumer data collection, and failed to meet regulatory requirements; the national health regulator subsequently ordered its removal.

Circumvention tactics were sparsely documented. *Product-based circumvention* included the introduction of newer products, such as e-cigarettes and smokeless tobacco, which were not anticipated when smoke-free regulations were first adopted and marketed as usable in environments covered by those regulations [221]. For example, in Mexico, e-cigarettes were popularised among youth and people who smoke for use in enclosed public places subject to smoke-free laws [121].

*Channel-based circumvention* was observed in India and Japan, where tobacco companies deployed mobile smoking vans that created temporary, industry-controlled smoking spaces at festivals and public locations [222]. These vans enabled smoking and cigarette sales in settings otherwise subject to smoke-free regulation.

Influencing implementation through intermediaries was the dominant tactic. Tobacco companies targeted *hospitality actors, enforcers, and public authorities*. In Mexico, corporate social responsibility campaigns misrepresented weaker national smoke-free provisions as overriding stronger local laws, encouraging restaurant and bar owners to establish designated smoking areas (DSAs) prohibited under local legislation [223]. Similarly, following Nepal’s ban on smoking in public places, the industry spread misinformation, contributing to non-compliance among businesses [17].

Enforcement actors were also targeted: in Nigeria, BAT was accused of misleading senior police officials about the definition of “public place” to deter enforcement [5], and in Japan, Japan Tobacco partnered with local governments to establish and manage outdoor DSAs, including through its “smoking manners” campaign following the adoption of municipal smoke-free ordinances [224, 225].

## Discussion

This scoping review is the first to systematically examine and categorise how the tobacco industry responds following policy adoption across four key tobacco control domains: TAPS, packaging and labelling, product-related regulation, and smoke-free measures. The findings show that the tobacco industry actively seeks to weaken adopted policies by undermining their effective implementation through a range of recurrent tactics. Together, these findings inform the development of an evidence-based, cross-policy taxonomy of post-adoption industry conduct, referred to here as the Policy Implementation Playbook (PIP).

The PIP identifies five broad tactics used by the tobacco industry. One tactic – pre-emptive adaptation – captures activities undertaken before a policy enters into force. Once a policy is in effect, the industry may disregard its requirements, adopt it in a tokenistic manner, or seek to circumvent it. Circumvention occurs through multiple mechanisms, including product-based, design-based, and channel-based strategies. Finally, the industry may seek to influence policy implementation indirectly through other actors, including retailers, hospitality actors, public authorities, and enforcers.

Across most policy areas, circumvention emerged as the dominant industry response, although its form varied by regulatory domain. Channel-based strategies were most common in response to TAPS policies, where promotional activity was displaced into less regulated environments such as PoS settings, online platforms, and social media. Packaging and labelling policies were more often met with design- and product-based adaptations intended to preserve brand meaning or reduce the salience or impact of misleading descriptor bans, health warnings, and standardised packaging requirements. Product-related policies frequently prompted the introduction of new or reclassified products that fell outside existing regulatory definitions.

Pre-emptive adaptation and disregard of policy requirements were documented across most policies, while token implementation was observed primarily in relation to health warning requirements. Efforts to shape implementation through intermediaries appeared proportionally more prominent in relation to smoke-free policies, possibly reflecting both the comparatively small evidence base and the central role of hospitality actors, local authorities, and enforcement agencies in implementing these measures. Taken together, these findings indicate that implementation is not merely technical but a politically contested phase during which the tobacco industry seeks to influence how regulations operate in practice.

Beyond policy-specific patterns, several cross-cutting dynamics were evident across regulatory domains. Where policies relied on product definitions or formal category boundaries, companies preserved cigarette-associated attributes either by modifying products or by shifting into adjacent regulatory categories, such as HTPs and separable components. As explicit promotional claims were restricted, brand meaning was increasingly embedded in product and pack design through colours, variant names, slogans, capsules, and filter innovations. Across policy domains, the PoS functioned as a key communication space, absorbing marketing and informational roles displaced from other channels, with retailer-mediated incentives and guidance shaping how restrictions operated in practice. Pre-emptive marketing intensification, stockpiling, and other transition activities further suggest that the period between policy adoption and entry into force represents a recurrent site of strategic industry adaptation.

In some cases, industry responses did not seek to undermine the adopted policy but instead redirected promotional activity into adjacent domains. For example, in anticipation of California’s 1998 smoke-free bar law, tobacco advertising intensified in San Francisco’s alternative press, suggesting a strategic shift in promotional focus rather than an attempt to undermine the smoke-free regulation directly [226].

Differences in the form of post-adoption industry responses across policy areas may reflect underlying differences in how these policies regulate tobacco marketing, products, and use. TAPS policies have typically developed incrementally, with advertising, promotion, and sponsorship channels restricted sequentially over time [227]. Such phased approaches repeatedly enabled promotional activity to shift into remaining or newly emerging channels. By contrast, comprehensive smoke-free policies, first introduced in California in 2003 and now adopted widely, regulate behaviour and space rather than product promotion, and have been associated with shifts in social norms around smoking [228]. These features appear to offer fewer opportunities for substitution and instead elicit responses focused on shaping interpretation, enforcement, and compliance.

The patterns identified in this review resonate with a growing literature on other unhealthy commodities. Recent work documents how marketing restrictions are circumvented in alcohol control [229], including through zero- and low-alcohol brand extensions that promote alcohol brands where marketing and sales are restricted [230]. Similar dynamics have been observed in relation to the International Code of Marketing of Breast-milk Substitutes, where product cross-promotion has been used to undermine the intent of regulation [231]. Concerns have also been raised in relation to the recent UK ‘junk food’ advertising ban, where companies may continue to promote brands despite restrictions on advertising specific products [232]. Beyond circumvention, the literature also documents instances of policy disregard in other industries [233] and highlights the regulation and enforcement of digital environments as a persistent cross-sector challenge [234, 235].

Taken together, this evidence suggests that corporate political activity is not only similar across sectors during policy formulation and adoption [15], but also during policy implementation. This reinforces the relevance of implementation-stage analysis for understanding how global corporate power is exercised through national regulatory systems, a core concern of the commercial determinants of health and global health governance literatures.

### Implications for policy design, implementation, and governance

After more than two decades of progress under the WHO FCTC [1] and the widespread adoption of tobacco control measures, the findings of this review underscore the need to give equal attention to policy implementation as a central site of contestation, alongside policy formulation and adoption. By making visible the recurrent patterns of post-adoption industry activity, the PIP provides a practical lens for anticipating likely tobacco industry responses and designing implementation arrangements that are more resilient to interference.

First, regulations that focus narrowly on specific products, channels, or formats appear particularly vulnerable to circumvention. Across policy areas, tobacco companies repeatedly exploited definitional boundaries, exemptions, and residual promotional spaces by reclassifying products, shifting into adjacent categories, or introducing new product forms and components. These findings suggest that effective regulation requires anticipatory scope and clear, robust definitions that minimise opportunities for substitution, including consideration of how new or hybrid tobacco and nicotine products may be positioned relative to existing regulatory categories.

Second, evidence from multiple policies indicates that regulatory effects may spill over into adjacent areas, as promotional activity displaced from one domain is redirected into others. For example, as explicit advertising claims were restricted, brand communication increasingly migrated into product and pack design or into retail environments. Designing policies with awareness of likely displacement pathways, informed by documented post-adoption industry responses, may help reduce unintended erosion of policy intent across regulatory domains.

Third, the industry activity between policy adoption and entry into force – including pre-emptive marketing intensification, stockpiling, and the use of transition materials – was observed across several policies, which indicates that phase-in arrangements and sell-off periods can weaken policy impact if not carefully managed. Treating implementation timelines as integral to regulatory design, rather than as administrative accommodations, may therefore be critical to maximising effectiveness.

Fourth, implementation outcomes were shaped not only by regulatory text but by the intermediaries through whom regulation is enacted. Across policy domains, tobacco companies sought to influence retailers, hospitality actors, public authorities, and enforcers through incentives, misinformation, or partnership arrangements. Clearer expectations, accountability mechanisms, and enforcement strategies directed at these intermediaries may be necessary to prevent erosion of policy intent during implementation.

Fifth, continued monitoring of post-adoption industry responses is essential, even in jurisdictions with comprehensive policies. As this review shows, tobacco companies continue to adapt their strategies over time, often in response to regulatory gaps and enforcement practices. Ongoing monitoring by regulators, civil society organisations, researchers, and international reporting mechanisms may be necessary to detect emerging post-adoption industry responses.

Finally, the persistence of cross-border tactics highlights the limits of isolated national approaches. Strengthening implementation guidance under the WHO FCTC, while applying similar anticipatory principles to the regulation of other unhealthy commodities, may help reduce opportunities for post-adoption erosion and reinforce efforts to address the commercial determinants of health.

### Strengths and limitations

This review has several strengths and limitations. First, it combined peer-reviewed literature with *Tobacco Control* *News Analysis* items, helping to mitigate the over-representation of high-income and Global North settings in the peer-reviewed literature, where research capacity, regulatory transparency, and civil society monitoring are typically stronger. Second, relatively strict eligibility criteria were applied, requiring explicit evidence or reasonably supported attribution of activities to the tobacco industry. While this ensured analytical clarity, it may have excluded some relevant practices where attribution could not be established, such as retailer non-compliance or activities by smoker-rights–style groups (e.g. [236]).

An important limitation is that the number of included sources reflects patterns of documentation rather than the frequency or intensity of post-adoption industry interference. This likely contributes to the predominance of findings related to TAPS policies and the comparatively limited documentation of post-adoption industry conduct in other domains, particularly smoke-free regulation. Source counts should therefore not be interpreted as indicators of how often interference occurs or its relative magnitude across policy domains or settings.

A further limitation is that relatively few sources documented industry efforts to influence implementation through intermediaries such as public authorities and enforcers, likely reflecting the less visible and harder-to-observe nature of these practices. In addition, this review focuses specifically on the tobacco industry and linked actors and does not capture the activities of actors without known industry links that may also undermine policy implementation.

The sources also did not allow for systematic analysis of responses to industry interference or their effectiveness. While some counterstrategies were described – particularly in *News Analysis *items – their outcomes were often unclear. Systematic research on post-adoption counterstrategies therefore represents an important area for future study, complementing existing work focusing on pre-adoption responses [237].

## Conclusion

This review demonstrates that tobacco industry interference does not end with policy adoption but continues through a range of tactics that can shape how regulations operate in practice. By conceptualising implementation as a contested political arena and synthesising industry activities across policy domains, the PIP extends existing frameworks of corporate political activity and highlights critical vulnerabilities in regulatory design and implementation. As tobacco control matures globally and comparable measures expand to other unhealthy commodities, the taxonomy offers a structured approach to anticipating and assessing post-adoption corporate conduct and to strengthening policy effectiveness across sectors.

## Electronic supplementary material

Below is the link to the electronic supplementary material.

Supplementary Material 1

## Data Availability

No datasets were generated or analysed during the current study.
