# Supplementary material for: The policy implementation playbook: a cross-policy taxonomy of post-adoption tobacco industry tactics
Source: Global Health. 2026 Jun 12;22:53. doi: 10.1186/s12992-026-01220-0 (PMC13281631; doi:10.1186/s12992-026-01220-0)
Supplement: Supplementary file 1 — Supplementary Material 1 [file 12992_2026_1220_MOESM1_ESM.pdf]

## Supplementary material

Table 1: List of included sources

| | Author | Year | Titel | Journal | News Analysis | Policy area* |
|---|---|---|---|---|---|---|
| **1** | Feinberg BM | 1971 | Content analysis shows cigarette advertising up twofold in 14 magazines | Journalism Quarterly | No | TAPS |
| **2** | Warner KE | 1985 | Tobacco industry response to a public health concern: A content analysis of cigarette ads | Health Education Quarterly | No | TAPS |
| **3** | Aitken P, Leathar D & Squair S | 1986 | Children's awareness of cigarette brand sponsorship of sports and games in the UK | Health Education Research | No | TAPS |
| **4** | Albright CL, Altman DG et al. | 1988 | Cigarette advertisements in magazines: Evidence for a differential focus on women's and youth magazines | Health Education Quarterly | No | TAPS |
| **5** | Blum A | 1991 | The Marlboro Grand Prix. Circumvention of the television ban on tobacco advertising | The New England Journal of Medicine | No | TAPS |
| **6** | King KW, Reid LN et al. | 1991 | Changes in the visual imagery of cigarette ads, 1954–1986 | Journal of Public Policy and Marketing | No | TAPS |
| **7** | AB | 1992 | Kuala Lumpur 1991 | Tobacco Control | Yes | TAPS |
| **8** | Robinson R, Barry M et al. | 1992 | Report of the Tobacco Policy Research Group on Marketing and Promotions Targeted at African Americans, Latinos, and Women | Tobacco Control | No | TAPS |
| **9** | AB & ES | 1993 | Canada leaving other countries out in the cold | Tobacco Control | Yes | TAPS |
| **10** | AB & ES | 1993 | Re-start your engines | Tobacco Control | Yes | TAPS |
| **11** | Bergman JE | 1993 | Tobacco battle takes to the sky | Tobacco Control | Yes | TAPS |
| **12** | Davis RM | 1993 | Vive la France | Tobacco Control | Yes | TAPS |
| **13** | Chapman S | 1994 | How astute a tobacco marketeer are you? | Tobacco Control | No | TAPS |
| **14** | Madden P & Grube J | 1994 | The frequency and nature of alcohol and tobacco advertising in televised sports, 1990 through 1992 | American Journal of Public Health | No | TAPS |
| **15** | Simpson D | 1994 | Camel burgers | Tobacco Control | Yes | TAPS |
| **16** | Simpson D | 1994 | Croatia: win some, lose some | Tobacco Control | Yes | TAPS |
| **17** | Simpson D | 1994 | Croatia: an extra war | Tobacco Control | Yes | TAPS |
| **18** | Hamann S | 1995 | Thailand survives art attack | Tobacco Control | Yes | TAPS |
| **19** | Simpson D | 1995 | China: Marlboro man evades ban | Tobacco Control | Yes | TAPS |
| **20** | Cornwell TB | 1997 | The use of sponsorship-linked marketing by tobacco firms: International public policy issues | The Journal of Consumer Affairs | No | TAPS |
| **21** | Jenkins CNH, Pham Xuan D et al. | 1997 | Tobacco use in Vietnam: Prevalence, predictors, and the role of the transnational tobacco corporations | JAMA | No | TAPS |
| **22** | Simpson D | 1997 | Turkey: Camel gets through again | Tobacco Control | Yes | TAPS |

**Supplementary material**

Table 1: List of included sources

| | | | | | | |
|---|---|---|---|---|---|---|
| **23** | Simpson D | 1997 | Turkey: driving a camel through the eye of the law | Tobacco Control | Yes | TAPS |
| **24** | Slade J | 1997 | The pack as advertisement | Tobacco Control | No | TAPS |
| **25** | Sparks R | 1997 | Tobacco-control Legislation, Public Health and Sport Sponsorship | Asia-Australia Marketing Journal | No | TAPS |
| **26** | Fraser T | 1998 | Phasing out of point-of-sale tobacco advertising in New Zealand | Tobacco Control | No | TAPS |
| **27** | Seimon T & Mehl GL | 1998 | Strategic marketing of cigarettes to young people in Sri Lanka: "go ahead--I want to see you smoke it now" | Tobacco Control | No | TAPS |
| **28** | Chaloupka F, Slater S & Wakefield M | 1999 | USA: price cuts and point of sale ads follow tax rise | Tobacco Control | Yes | TAPS |
| **29** | Simpson D | 1999 | Turkey: tobacco's hard drive for ban-busting F1 | Tobacco Control | Yes | TAPS |
| **30** | Simpson D | 1999 | South Africa: two winnings and a funeral boom | Tobacco Control | Yes | TAPS |
| **31** | Efroymson D | 2000 | Bangladesh: voyage of disdain sunk without trace | Tobacco Control | Yes | TAPS |
| **32** | Vateesatokit P, Hughes B & Ritthphakdee B | 2000 | Thailand: winning battles, but the war's far from over | Tobacco Control | No | TAPS |
| **33** | Carter S | 2001 | Worshipping at the Alpine altar: promoting tobacco in a world without advertising | Tobacco Control | No | TAPS |
| **34** | Chantornvong S & McCargo D | 2001 | Political economy of Tobacco Control in Thailand | Tobacco Control | No | TAPS |
| **35** | Feighery EC, Ribisl KM et al. | 2001 | Cigarette advertising and promotional strategies in retail outlets: results of a statewide survey in California | Tobacco Control | No | TAPS |
| **36** | Harper T | 2001 | Marketing life after advertising bans | Tobacco Control | No | TAPS |
| **37** | King C & Siegel M | 2001 | The Master Settlement Agreement with the tobacco industry and cigarette advertising in magazines | The New England Journal of Medicine | No | TAPS |
| **38** | Oddouz K & Melihan-Cheinin P | 2001 | France: rolling round the curbs | Tobacco Control | Yes | TAPS |
| **39** | Rosenberg NJ & Siegel M | 2001 | Use of corporate sponsorship as a tobacco marketing tool: a review of tobacco industry sponsorship in the USA, 1995–99 | Tobacco Control | No | TAPS |
| **40** | Smith KC & Wakefield M | 2001 | USA: the name of Philip Morris to sit on 28 million school desks | Tobacco Control | Yes | TAPS |
| **41** | Anderson S, Hastings G & MacFadyen L | 2002 | Strategic marketing in the UK tobacco industry | The Lancet Oncology | No | TAPS |
| **42** | Assunta M | 2002 | BAT flouts tobacco-free World Cup policy | Tobacco Control | No | TAPS |

**Supplementary material**

Table 1: List of included sources

| | | | | | | |
|---|---|---|---|---|---|---|
| **43** | Celebucki CC & Diskin K | 2002 | A longitudinal study of externally visible cigarette advertising on retail storefronts in Massachusetts before and after the Master Settlement Agreement | Tobacco Control | No | TAPS |
| **44** | Dewhirst T & Hunter A | 2002 | Tobacco sponsorship of Formula One and CART auto racing: tobacco brand exposure and enhanced symbolic imagery through co-sponsors' third party advertising | Tobacco Control | No | TAPS |
| **45** | Hamilton WL, Turner-Bowker C et al. | 2002 | Cigarette advertising in magazines: the tobacco industry response to the Master Settlement Agreement and to public pressure | Tobacco Control | No | TAPS |
| **46** | Harper TA & Martin JE | 2002 | Under the radar-how the tobacco industry targets youth in Australia | Drug and Alcohol Review | No | TAPS |
| **47** | Sepe E, Ling PM & Glantz SA | 2002 | Smooth moves: Bar and nightclub tobacco promotions that target young adults | American Journal of Public Health | No | TAPS |
| **48** | Simpson D | 2002 | Hong Kong: Marlboro tries it on (the pack) | Tobacco Control | Yes | TAPS |
| **49** | Simpson D | 2002 | Thailand: trying to swing it on the golf course | Tobacco Control | Yes | TAPS |
| **50** | Wakefield M & Letcher T | 2002 | My pack is cuter than your pack | Tobacco Control | No | TAPS |
| **51** | Wakefield M, Morley C et al. | 2002 | The cigarette pack as image: new evidence from tobacco industry documents | Tobacco Control | No | TAPS |
| **52** | Wakefield MA, Terry-McElrath YM et al. | 2002 | Tobacco industry marketing at point of purchase after the 1998 MSA billboard advertising ban | American Journal of Public Health | No | TAPS |
| **53** | Carter SM | 2003 | Going below the line: creating transportable brands for Australia's dark market | Tobacco Control | No | TAPS |
| **54** | Carter SM | 2003 | New frontier, new power: the retail environment in Australia's dark market | Tobacco Control | No | TAPS |
| **55** | Chebat JC | 2003 | Effects of F1 grand prix sponsorship by cigarette brands on adolescents' cognitive and behavioral responses | International Journal of Sports Marketing and Sponsorship | No | TAPS |
| **56** | Greaves L | 2003 | Canada: demolishing the power walls | Tobacco Control | Yes | TAPS |
| **57** | Lavack AM | 2003 | An inside view of tobacco sports sponsorship: an historical perspective | International Journal of Sports Marketing and Sponsorship | No | TAPS |
| **58** | Morrow M & Barraclough S | 2003 | Tobacco Control and gender in Southeast Asia. Part I: Malaysia and the Philippines | Health Promotion International | No | TAPS |
| **59** | Simpson D | 2003 | UK: getaway cars? | Tobacco Control | Yes | TAPS |
| **60** | Assunta M & Chapman S | 2004 | "The world's most hostile environment": how the tobacco industry circumvented Singapore's advertising ban | Tobacco Control | No | TAPS |
| **61** | Assunta M & Chapman S | 2004 | The tobacco industry's accounts of refining indirect tobacco advertising in Malaysia | Tobacco Control | No | TAPS |

**Supplementary material**

Table 1: List of included sources

| | | | | | | |
|---|---|---|---|---|---|---|
| **62** | Assunta M, Chapman S | 2004 | Industry sponsored youth smoking prevention programme in Malaysia: a case study in duplicity | Tobacco Control | No | TAPS |
| **63** | Biener L, Nyman AL et al. | 2004 | Adults only: The prevalence of tobacco promotions in bars and clubs in the Boston Area | Tobacco Control | No | TAPS |
| **64** | Braverman MT & Aarø LE | 2004 | Adolescent smoking and exposure to tobacco marketing under a tobacco advertising ban: findings from 2 Norwegian national samples | American Journal of Public Health | No | TAPS |
| **65** | Carlyle J, Collin J et al. | 2004 | British American Tobacco and Formula One motor racing | BMJ | No | TAPS |
| **66** | Knight J & Chapman S | 2004 | "Asian yuppies ... are always looking for something new and different": creating a tobacco culture among young Asians | Tobacco Control | No | TAPS |
| **67** | Knowles JH, Wanke KL & Kawachi I | 2004 | Internet sales of tobacco: heading off the new E-pidemic | Journal of Public Health Policy | No | TAPS |
| **68** | Kohrman M | 2004 | Should I Quit? Tobacco, Fraught Identity, And the Risks of Governmentality In Urban China | Urban Anthropology and Studies of Cultural Systems and World Economic Development | No | TAPS |
| **69** | Lee K, Gilmore AB & Collin J | 2004 | Breaking and re-entering: British American Tobacco in China 1979-2000 | Tobacco Control | No | TAPS |
| **70** | MacKenzie R, Collin J et al. | 2004 | "If we can just 'stall' new unfriendly legislations, the scoreboard is already in our favour": transnational tobacco companies and ingredients disclosure in Thailand | Tobacco Control | No | P-R |
| **71** | Ruel E, Mani N et al. | 2004 | After the Master Settlement Agreement: trends in the American tobacco retail environment from 1999 to 2002 | Health Promotion Practice | No | TAPS |
| **72** | Shatenstein S | 2004 | Canada: chicanery in the chicanes | Tobacco Control | Yes | TAPS |
| **73** | Simpson D | 2004 | USA/Brazil: the flavour of things to come? | Tobacco Control | Yes | P&L, TAPS |
| **74** | Simpson D | 2004 | Japan, India: mobile smoking vans | Tobacco Control | Yes | SF |
| **75** | Simpson D | 2004 | UK: how did these get here? | Tobacco Control | Yes | TAPS |
| **76** | Simpson D | 2004 | Malaysia: racing round the hurdles | Tobacco Control | Yes | TAPS |
| **77** | Simpson D | 2004 | USA: they were putting what, where? | Tobacco Control | Yes | TAPS |
| **78** | Simpson D | 2004 | Brazil: BAT's "smoking point" banned | Tobacco Control | Yes | SF |
| **79** | Szilagyi T | 2004 | Hungary: tobacco ads forced back inside | Tobacco Control | Yes | TAPS |
| **80** | Bansal R, John S & Ling PM | 2005 | Cigarette advertising in Mumbai, India: targeting different socioeconomic groups, women, and youth | Tobacco Control | No | TAPS |
| **81** | Gilpin EA, White VM & Pierce JP | 2005 | How effective are tobacco industry bar and club marketing efforts in reaching young adults? | Tobacco Control | No | TAPS |

## Supplementary material
Table 1: List of included sources

| | | | | | | |
|---|---|---|---|---|---|---|
| **82** | Kaduri P, Gilreath T et al. | 2005 | Social networks' influence on tobacco use among students in Dar Es Salaam, Tanzania | Promotion & Education | No | TAPS |
| **83** | King B & Borland R | 2005 | What was ‘‘light’’ and ‘‘mild’’ is now ‘‘smooth’’ and ‘‘fine’’: new labelling of Australian cigarettes | Tobacco Control | No | P&L |
| **84** | Mehl G, Wipfli H & Winch P | 2005 | Controlling Tobacco: The Vital Role of Local Communities | Harvard International Review | No | TAPS |
| **85** | Simpson D | 2005 | India: still at the bravery game | Tobacco Control | Yes | TAPS |
| **86** | Simpson D | 2005 | Swedish Match: sucked into controversy, worldwide | Tobacco Control | Yes | P&L, TAPS |
| **87** | Simpson D | 2005 | China: illegal brand ads pulled | Tobacco Control | Yes | TAPS |
| **88** | Simpson D | 2005 | Hungary: BAT university deal questioned | Tobacco Control | Yes | TAPS |
| **89** | Snell C & Bailey L | 2005 | Operation storefront: observations of tobacco retaller advertising and compliance with tobacco laws | Youth Violence and Juvenile Justice | No | TAPS |
| **90** | Sushma C & Sharang C | 2005 | Pan masala advertisements are surrogate for tobacco products | Indian Journal of Cancer | No | TAPS |
| **91** | Wen, CP, Chen T et al. | 2005 | Are marketing campaigns in Taiwan by foreign tobacco companies targeting young smokers? | Tobacco Control | No | TAPS |
| **92** | Goswami H | 2006 | India: promoting tobacco via “research” | Tobacco Control | Yes | TAPS |
| **93** | Hafez N & Ling PM | 2006 | Finding the Kool Mixx: how Brown & Williamson used music marketing to sell cigarettes | Tobacco Control | No | TAPS |
| **94** | Hammond D | 2006 | Canada: a new angle on packs | Tobacco Control | Yes | TAPS |
| **95** | Jalleh G, Donovan RJ et al. | 2006 | Tobacco advertising and promotion at point-of-sale | Australian and New Zealand Journal of Public Health | No | P&L, TAPS |
| **96** | Lavack AM & Toth G | 2006 | Tobacco point-of-purchase promotion: examining tobacco industry documents | Tobacco Control | No | TAPS |
| **97** | Loomis BR, Farrelly MC et al. | 2006 | Point of purchase cigarette promotions before and after the Master Settlement Agreement: exploring retail scanner data | Tobacco Control | No | TAPS |
| **98** | Simpson D | 2006 | India: oral tobacco kite ads | Tobacco Control | Yes | TAPS |
| **99** | Simpson D | 2006 | Australia: BAT corporate social irresponsibility | Tobacco Control | Yes | P&L |
| **100** | Simpson D | 2006 | Czech Republic: film targets still in frame | Tobacco Control | Yes | TAPS |
| **101** | Simpson D | 2006 | Hong Kong, China: Marlboro pack sleeves | Tobacco Control | Yes | P&L |
| **102** | Swanson MG | 2006 | Australia: health warnings canned | Tobacco Control | Yes | P&L |
| **103** | Chapman S | 2007 | Australia: British American Tobacco “addresses” youth smoking | Tobacco Control | Yes | P&L |

## Supplementary material

Table 1: List of included sources

| | | | | | | |
|---|---|---|---|---|---|---|
| **104** | Chaudhry S, Chaudhry S & Chaudhry K | 2007 | Point of sale tobacco advertisements in India | Indian Journal of Cancer | No | TAPS |
| **105** | Delnevo CD & Hrywna M | 2007 | "A whole 'nother smoke" or a cigarette in disguise: how RJ Reynolds reframed the image of little cigars | American Journal of Public Health | No | P&L, TAPS |
| **106** | Kralikova E | 2007 | Czech Republic: BAT gets round weak ad ban | Tobacco Control | Yes | TAPS |
| **107** | MacKenzie R, Collin J & Sriwongcharoen K | 2007 | Thailand - lighting up a dark market: British American tobacco, sports sponsorship and the circumvention of legislation | Journal of Epidemiology and Community Health | No | TAPS |
| **108** | Sanders S | 2007 | Australia: WARNING: outdated pack health warnings are addictive - to tobacco companies | Tobacco Control | Yes | P&L |
| **109** | Simpson D | 2007 | Belgium: retail incentives are child's play | Tobacco Control | Yes | TAPS |
| **110** | Simpson D | 2007 | UK: FI promotion dead, but won't lie down | Tobacco Control | Yes | TAPS |
| **111** | Simpson D | 2007 | South Korea: pack design bombshell | Tobacco Control | Yes | TAPS |
| **112** | Slater SJ, Chaloupka FJ et al. | 2007 | The Impact of Retail Cigarette Marketing Practices on Youth Smoking Uptake | Archives of Pediatrics and Adolescent Medicine | No | TAPS |
| **113** | Sussman S, Pokhrel P et al. | 2007 | Tobacco control in developing countries: Tanzania, Nepal, China, and Thailand as examples | Nicotine & Tobacco Research | No | P&L |
| **114** | Alpert HR, Koh HK & Connolly GN | 2008 | After The Master Settlement Agreement: Targeting And Exposure Of Youth To Magazine Tobacco Advertising | Health Affairs | No | TAPS |
| **115** | Borland R, Fong GT et al. | 2008 | What Happened to Smokers' Beliefs about Light Cigarettes When "Light/Mild" Brand Descriptors Were Banned in the UK? Findings from the International Tobacco Control (ITC) Four Country Survey | Tobacco Control | No | P&L |
| **116** | Feighery EC, Schleicher NC et al. | 2008 | An Examination of Trends in Amount and Type of Cigarette Advertising and Sales Promotions in California Stores, 2002-2005 | Tobacco Control | No | TAPS |
| **117** | Freeman B, Chapman S & Rimmer M | 2008 | The case for the plain packaging of tobacco products | Addiction | No | P&L |
| **118** | Lee S & Holden C | 2008 | South Korea: KT&G prepares for FCTC with charity drive | Tobacco Control | Yes | TAPS |
| **119** | Lee S & Lee K | 2008 | South Korea: Smoke on the Roads | Tobacco Control | Yes | TAPS |
| **120** | Mackenzie R | 2008 | Australia: Don't Mention the Product | Tobacco Control | Yes | TAPS |
| **121** | Saloojee Y | 2008 | South Africa: Snus "not a tobacco product" | Tobacco Control | Yes | TAPS |

## Supplementary material
Table 1: List of included sources

| | | | | | | |
|---|---|---|---|---|---|---|
| **122** | Scheffels, J | 2008 | "A difference that makes a difference: young adult smokers' accounts of cigarette brands and package design" | Tobacco Control | No | TAPS |
| **123** | Simpson D | 2008 | Philippines: Bad Year for PM's PR | Tobacco Control | Yes | TAPS |
| **124** | Simpson D | 2008 | Philippines: String of Marlboro Packs | Tobacco Control | Yes | P&L |
| **125** | Sinha, DN, Gupta PC et al. | 2008 | Linking Global Youth Tobacco Survey 2003 and 2006 data to tobacco control policy in India | Journal of School Health | No | TAPS |
| **126** | Danishevskiy K & Saverskiy A | 2009 | Russia: Pandora's box, or holy fool's flash mob? | Tobacco Control | Yes | TAPS |
| **127** | Freeman B & Chapman S | 2009 | Open-source marketing: Camel cigarette brand marketing in the "Web 2.0" world | Tobacco Control | No | TAPS |
| **128** | John R, Cheney MK & Azad MR | 2009 | Point-of-sale marketing of tobacco products: taking advantage of the socially disadvantaged? | Journal of Health Care for the Poor and Underserved | No | TAPS |
| **129** | Lee K | 2009 | Russia: What is JTI playing at? | Tobacco Control | Yes | TAPS |
| **130** | Moodie C & Hastings G | 2009 | Making the pack the hero, tobacco industry response to marketing restrictions in the UK: findings from a long-term audit | International Journal of Mental Health & Addiction | No | TAPS |
| **131** | Peace J, Wilson N et al. | 2009 | Survey of descriptors on cigarette packs: still misleading consumers? | The New Zealand Medical Journal | No | P&L |
| **132** | Preechawong S | 2009 | Thailand: Wiping out the last ads | Tobacco Control | Yes | P&L |
| **133** | Simpson D | 2009 | India: Surrogates and spoofs | Tobacco Control | Yes | TAPS |
| **134** | Simpson D | 2009 | Sri Lanka: Industry promotion | Tobacco Control | Yes | TAPS |
| **135** | Simpson D | 2009 | Switzerland: Imperial's tennis racket | Tobacco Control | Yes | TAPS |
| **136** | Wilson N, Peace J et al. | 2009 | Distribution of new graphic warning labels: Are tobacco companies following regulations? | Tobacco Induced Diseases | No | P&L |
| **137** | Elkin L, Thomson G & Wilson N | 2010 | Connecting world youth with tobacco brands YouTube and the Internet policy vacuum on Web 2.0 | Tobacco Control | No | TAPS |
| **138** | Freeman B & Chapman S | 2010 | British American Tobacco on Facebook: undermining Article 13 of the global World Health Organization Framework Convention on Tobacco Control | Tobacco Control | No | TAPS |
| **139** | Hoek J, Wilson N et al. | 2010 | Lessons from New Zealand's introduction of pictorial health warnings on tobacco packaging | Bulletin of the World Health Organization | No | P&L |
| **140** | Kin F, Lian TY & Yoon YC | 2010 | How the Tobacco Industry Circumvented Ban on Tobacco Advertising, Promotion and Sponsorship: Observations from Selected ASEAN Countries | Asian Journal of WTO & International Health Law and Policy | No | TAPS |
| **141** | King B, Borland R et al. | 2010 | Divergence between strength indicators in packaging and cigarette engineering: a case study of Marlboro varieties in Australia and the USA | Tobacco Control | No | P&L |

## Supplementary material

Table 1: List of included sources

| | | | | | | |
|---|---|---|---|---|---|---|
| **142** | Longman JM, Pritchard C et al. | 2010 | Accessibility of chewing tobacco products in England | Journal of Public Health | No | P&L |
| **143** | McKnight-Eily L, Arrazola R et al. | 2010 | Prevalence and Psychosocial Correlates of Current Smoking Among Adolescent Students in Thailand, 2005 | Health Education & Behavior | No | TAPS |
| **144** | Moodie C & Hastings G | 2010 | Tobacco packaging as promotion | Tobacco Control | No | TAPS |
| **145** | Rodriguez-Bolanos R, Reynales-Shigematsu LM et al. | 2010 | Monitoring strategy for control of tobacco in Mexico: advertising, promotion and sponsorship, packaging and labeling | Salud Pública de México | No | P&L, TAPS |
| **146** | Rooke C, Cheeseman H et al. | 2010 | Tobacco point-of-sale displays in England: a snapshot survey of current practices | Tobacco Control | No | TAPS |
| **147** | Sebrie EM, Blanco A & Glantz SA | 2010 | Cigarette labeling policies in Latin America and the Caribbean: progress and obstacles | Salud Pública de México | No | P&L |
| **148** | Simpson D | 2010 | Switzerland: Davidoff loses game, set &match | Tobacco Control | Yes | TAPS |
| **149** | Simpson D | 2010 | Mauritius: Women in politics the BAT link | Tobacco Control | Yes | TAPS |
| **150** | Simpson D | 2010 | Germany: BAT'S political ad | Tobacco Control | Yes | TAPS |
| **151** | Simpson D | 2010 | Switzerland: Health beats Davidoff beats Davidoff | Tobacco Control | Yes | TAPS |
| **152** | Thrasher JF, Hammond D & Arillo-Santillán E | 2010 | The alchemy of Marlboro: transforming 'light' into 'gold' in Mexico | Tobacco Control | No | P&L |
| **153** | Bansal-Travers M, Hammond D et al. | 2011 | The Impact of Cigarette Pack Design, Descriptors, and Warning Labels on Risk Perception in the U.S. | American Journal of Preventive Medicine | No | P&L |
| **154** | Bansal-Travers M, O'Connor R et al. | 2011 | What do cigarette pack colors communicate to smokers in the U.S.? | American Journal of Preventive Medicine | No | P&L |
| **155** | Coombs J, Bond L et al. | 2011 | "Below the Line": The tobacco industry and youth smoking | Australasian Medical Journal | No | TAPS |
| **156** | Crosbie E, Sebrie E & Glantz S | 2011 | Strong advocacy led to successful implementation of smokefree Mexico City | Tobacco Control | No | SF |
| **157** | Danishevskiy K & McKee M | 2011 | Tobacco: a product like any other? | Health Economics, Policy and Law | No | P&L |

# Supplementary material
## Table 1: List of included sources

| | | | | | | |
|---|---|---|---|---|---|---|
| **158** | Mir H, Buchanan D et al. | 2011 | Cigarette pack labelling in 12 countries at different levels of economic development | Journal of Public Health Policy | No | P&L |
| **159** | Mutti S, Hammond D et al. | 2011 | Beyond light and mild: cigarette brand descriptors and perceptions of risk in the International Tobacco Control (ITC) Four Country Survey | Addiction | No | P&L |
| **160** | Nimpitakpong P & Pittayakulmongkon C | 2011 | Thailand: Young, female 'Ambassadors' promote cigarettes | Tobacco Control | Yes | TAPS |
| **161** | Siahpush M, Borland R et al. | 2011 | Socioeconomic differences in the effectiveness of the removal of the "light" descriptor on cigarette packs: findings from the International Tobacco Control (ITC) Thailand Survey | International Journal of Environmental Research and Public Health | No | P&L |
| **162** | Simpson D | 2011 | World: New Marlboro F1 sponsorship but why? | Tobacco Control | Yes | TAPS |
| **163** | Stanton CR, Chu A et al. | 2011 | Promoting tobacco through the international language of dance music: British American Tobacco and the Ministry of Sound | European Journal of Public Health | No | TAPS |
| **164** | Tumwine J | 2011 | Implementation of the Framework Convention on Tobacco Control in Africa: Current Status of Legislation | International Journal of Environmental Research and Public Health | No | P&L, TAPS |
| **165** | Charoenca N, Mock J et al. | 2012 | Success Counteracting Tobacco Company Interference in Thailand: An Example of FCTC Implementation for Low- and Middle-income Countries | International Journal of Environmental Research and Public Health | No | TAPS |
| **166** | Islam Z | 2012 | Pakistan: Legal action against Philip Morris for advertising ban violation | Tobacco Control | Yes | TAPS |
| **167** | Nair S, Schensul JJ et al. | 2012 | Local responses to the Maharashtra gutka and pan masala ban: a report from Mumbai | Indian Journal of Cancer | No | P-R |
| **168** | Simpson D | 2012 | Lebanon: Testing time as new ad ban takes effect | Tobacco Control | Yes | TAPS |
| **169** | Simpson D | 2012 | India/Australia: Row over oral tobacco cricket ads | Tobacco Control | Yes | TAPS |
| **170** | Simpson D | 2012 | Cambodia: KT&G? 'Korean tomorrow & Global' of course | Tobacco Control | Yes | TAPS |
| **171** | Simpson D | 2012 | New Zealand: Pack seal can cover warnings | Tobacco Control | Yes | P&L |
| **172** | Tan YL & Foong K | 2012 | How the Malaysian tobacco industry exploits loopholes in pictorial health warnings | Tobacco Control | No | P&L, TAPS |
| **173** | Wakefield M, Zacher M et al. | 2012 | Brand placement on price boards after tobacco display bans: a point-of-sale audit in Melbourne, Australia | Tobacco Control | No | TAPS |
| **174** | Britton J & Bogdanovica I | 2013 | Tobacco control efforts in Europe | The Lancet | No | TAPS |
| **175** | Kashiwabara M & Armada F | 2013 | Mind your "smoking manners": The tobacco industry tactics to normalize smoking in Japan | Kobe Journal of Medical Sciences | No | SF |

**Supplementary material**

Table 1: List of included sources

| | | | | | | |
|---|---|---|---|---|---|---|
| **176** | Kotnowski K & Hammond D | 2013 | The impact of cigarette pack shape, size and opening: evidence from tobacco company documents | Addiction | No | TAPS |
| **177** | Mir H, Roberts B et al. | 2013 | Analysing compliance of cigarette packaging with the FCTC and national legislation in eight former Soviet countries | Tobacco Control | No | P&L |
| **178** | Purpera J | 2013 | USA: Cigarette packs legal tussle | Tobacco Control | Yes | P&L |
| **179** | Purpera J | 2013 | Korea: JTI targeting young people | Tobacco Control | Yes | TAPS |
| **180** | Rathinam A | 2013 | India: Disrupting tobacco industry CSR in Chennai | Tobacco Control | Yes | TAPS |
| **181** | Schensul JJ, Nair S et al. | 2013 | Availability, accessibility and promotion of smokeless tobacco in a low-income area of Mumbai | Tobacco Control | No | TAPS |
| **182** | van Walbeek C, Blecher E et al. | 2013 | Price and Tax Measures and Illicit Trade in the Framework Convention on Tobacco Control: What We Know and What Research Is Required | Nicotine & Tobacco Research | No | P-R |
| **183** | Burton S, Williams K et al. | 2014 | Marketing cigarettes when all else is unavailable: Evidence of discounting in price-sensitive neighborhoods | Tobacco Control | No | TAPS |
| **184** | Cohen JE, Yang J & Donaldson EA | 2014 | Impact of the removal of light and mild descriptors from cigarette packages in Ontario, Canada: Switching to “light replacement” brand variants | Preventive Medicine | No | P&L |
| **185** | Connolly GN & Alpert HR | 2014 | Has the tobacco industry evaded the FDA's ban on 'Light' cigarette descriptors? | Tobacco Control | No | P&L |
| **186** | Goel S, Kumar R et al. | 2014 | How compliant are tobacco vendors to India’s tobacco control legislation on Ban of advertisments at point of sale? A three jurisdictions review | Asian Pacific Journal of Can cer Prevention | No | TAPS |
| **187** | Hefler M | 2014 | Brazil/ German: Be Malboro, Be Fined, Be Banned | Tobacco Control | Yes | TAPS |
| **188** | Hefler M | 2014 | Indonesia: Tobacco companies flout pictorial laws | Tobacco Control | Yes | P&L |
| **189** | Hefler M | 2014 | World: Philip Morris campaign targets teens | Tobacco Control | Yes | TAPS |
| **190** | Maziak W, Nakkash R et al. | 2014 | Tobacco in the Arab world: old and new epidemics amidst policy paralysis | Health Policy and Planning | No | TAPS |
| **191** | Moodie C, Angus K & Ford A | 2014 | The importance of cigarette packaging in a ’dark’ market: the ’Silk Cut’ experience | Tobacco Control | No | TAPS |
| **192** | Tan YL & Foong K | 2014 | Tobacco industry tangos with descriptor ban in Malaysia | Tobacco Control | No | P&L |
| **193** | Wang F, Zheng P et al. | 2014 | Chinese tobacco industry promotional activity on the microblog Weibo | PLoS One | No | TAPS |
| **194** | Zheng PP, Ge X et al. | 2014 | Zhonghua' tobacco advertisement in Shanghai: a descriptive study | Tobacco Control | No | TAPS |
| **195** | Baker J | 2015 | World: Marlboro & Ferrari continue F1 partnership | Tobacco Control | Yes | TAPS |
| **196** | Beaglehole R, Bonita R et al. | 2015 | A tobacco-free world: A call to action to phase out the sale of tobacco products by 2040 | The Lancet | No | P-R |

**Supplementary material**

Table 1: List of included sources

| | | | | | | |
|---|---|---|---|---|---|---|
| **197** | Chaturvedi P & Seth S | 2015 | India: Smokeless Tobacco ban shows early success | Tobacco Control | Yes | P-R |
| **198** | Delnevo CD & Hrywna M | 2015 | Clove cigar sales following the US flavoured cigarette ban | Tobacco Control | No | P-R |
| **199** | Elton-Marshall T, Fong, GT et al. | 2015 | Smokers' sensory beliefs mediate the relation between smoking a light/low tar cigarette and perceptions of harm | Tobacco Control | No | P&L |
| **200** | Gilmore AB, Fooks G et al. | 2015 | Exposing and addressing tobacco industry conduct in low-income and middle-income countries | The Lancet | No | P&L, SF |
| **201** | Hefler M | 2015 | World: Be Marlboro, be invisible | Tobacco Control | Yes | TAPS |
| **202** | Hefler M | 2015 | Bangladesh: Tobacco-sponsored concerts cancelled | Tobacco Control | Yes | TAPS |
| **203** | Scollo M, Occleston J et al. | 2015 | Tobacco product developments coinciding with the implementation of plain packaging in Australia | Tobacco Control | No | P&L |
| **204** | Soerojo W | 2015 | Indonesia: Warnings need stronger enforcement | Tobacco Control | Yes | P&L |
| **205** | White V, Bariola E et al. | 2015 | Graphic Health Warnings on Cigarette Packs | Nicotine & Tobacco Research | No | P&L |
| **206** | Arevalo R, Corral J et al. | 2016 | Characteristics of illegal and legal cigarette packs sold in Guatemala | Globalization and Health | No | P&L |
| **207** | Cohen JE, Brown J et al. | 2016 | Do cigarette health warning labels comply with requirements: A 14-country study | Preventive Medicine | No | P&L |
| **208** | Eadie D, Stead M et al. | 2016 | Are Retail Outlets Complying with National Legislation to Protect Children from Exposure to Tobacco Displays at Point of Sale? Results from the First Compliance Study in the UK | Plos One | No | TAPS |
| **209** | Greenland SJ | 2016 | The Australian experience following plain packaging: the impact on tobacco branding | Addiction | No | P&L |
| **210** | Greenland SJ, Johnson, L & Seifi S | 2016 | Tobacco manufacturer brand strategy following plain packaging in Australia: implications for social responsibility and policy | Social Responsibility Journal | No | P&L |
| **211** | Hefler M | 2016 | The Philippines: Tobacco companies exploit transition to graphic health warnings | Tobacco Control | Yes | P&L |
| **212** | Kostygina G, Glantz SA & Ling PM | 2016 | Tobacco industry use of flavours to recruit new users of little cigars and cigarillos | Tobacco Control | No | P-R |
| **213** | Liutkutė V | 2016 | Lithuania: FCTC breaches undermine tobacco control progress | Tobacco Control | Yes | TAPS |
| **214** | Smith K, Washington C et al. | 2016 | Cigarette stick as valuable communicative real estate: a content analysis of cigarettes from 14 low-income and middle-income countries | Tobacco Control | No | TAPS |
| **215** | Vidhubala E, Pisinger C et al. | 2016 | The ban on smokeless tobacco products is systematically violated in Chennai, India | Indian Journal of Cancer | No | P-R |

**Supplementary material**

Table 1: List of included sources

| | | | | | | |
|---|---|---|---|---|---|---|
| **216** | Yun EH, Park YO et al. | 2016 | Public opinions on disclosure of tobacco components Results of a nationwide cross-sectional survey in Republic of Korea | Medicine | No | SF |
| **217** | African Population and Health Research Center | 2017 | 5.0 Case Study 2 – Application of Multi-sectoral Action in Tobacco Control Policies | Analysis of Non-Communicable Disease Prevention Policies in South Africa | No | TAPS |
| **218** | Brown J, DeAtley T et al. | 2017 | Tobacco industry response to menthol cigarette bans in Alberta and Nova Scotia, Canada | Tobacco Control | No | P-R |
| **219** | Egbe CO, Bialous SA & Glantz SA | 2017 | Avoiding "a Massive Spin-Off Effect in West Africa and Beyond": The Tobacco Industry Stymies Tobacco Control in Nigeria | Nicotine & Tobacco Research | No | TAPS |
| **220** | Hefler M | 2017 | India: Philip Morris' bold flouting of law exposed | Tobacco Control | Yes | TAPS |
| **221** | Hefler M | 2017 | UK: last-ditch attempt to undermine plain packs | Tobacco Control | Yes | P&L |
| **222** | Kennedy RD, Grant A et al. | 2017 | Point-of-sale Tobacco Advertising and Display Bans: Policy Evaluation Study in Five Russian Cities | JMIR Public Health and Surveillance | No | TAPS |
| **223** | MacKenzie R & Collin J | 2017 | A preferred consultant and partner to the Royal Government, NGOs, and the community': British American Tobacco's access to policy-makers in Cambodia | Global Public Health | No | TAPS |
| **224** | Alpert HR, Carpenter D & Connolly GN | 2018 | Tobacco industry response to a ban on lights descriptors on cigarette packaging and population outcomes | Tobacco Control | No | P&L |
| **225** | Astuti PAS, Assunta M & Freeman B | 2018 | Raising generation 'A': a case study of millennial tobacco company marketing in Indonesia | Tobacco Control | No | TAPS |
| **226** | Breton MO, Britton J et al. | 2018 | Cigarette brand diversity and price changes during the implementation of plain packaging in the United Kingdom | Addiction | No | P&L |
| **227** | Critchlow N, Stead M et al. | 2018 | Introduction of standardised tobacco packaging during a 12-month transition period: Findings from small retailers in the United Kingdom | Nicotine & Tobacco Research | No | P&L |
| **228** | Hefler M | 2018 | UK: PMI new 'premium smoking experience' launched in key 'smoke-free future' market | Tobacco Control | Yes | P&L |
| **229** | Kass Lempert L & Glantz SA | 2018 | Heated tobacco product regulation under US law | Tobacco Control | No | P&L, TAPS |
| **230** | Kim M | 2018 | Philip Morris International introduces new heat-not-burn product, IQOS, in South Korea | Tobacco Control | No | P&L |
| **231** | Mathers A, Schwartz R et al. | 2018 | Marketing IQOS in a dark market | Tobacco Control | No | TAPS |
| **232** | McDaniel PA, Cadman B & Malone RE | 2018 | African media coverage of tobacco industry corporate social responsibility initiatives | Global Public Health | No | TAPS |

## Supplementary material

Table 1: List of included sources

| | | | | | | |
|---|---|---|---|---|---|---|
| **233** | Mohamed SF, Juma P et al. | 2018 | Facilitators and barriers in the formulation and implementation of tobacco control policies in Kenya: a qualitative study | BMC Public Health | No | TAPS |
| **234** | Moodie C, Angus K & Mitchell D | 2018 | How tobacco companies in the United Kingdom prepared for, and responded to, standardised packaging of cigarettes and rolling tobacco | Tobacco Control | No | P&L |
| **235** | Moodie C, Hoek J et al. | 2018 | Plain packaging: legislative differences in Australia, France, the UK, New Zealand and Norway, and options for strengthening regulations | Tobacco Control | No | P&L |
| **236** | Nyi Latt N, Saw YM et al. | 2018 | Tobacco control law awareness, enforcement, and compliance among high school students in Myanmar | Nagoya Journal of Medical Science | No | TAPS |
| **237** | Saraf DS, Mehrotra R et al. | 2018 | A review of trade practices of smokeless tobacco products in terms of prohibition on sale, manufacturing & Importation in Framework Convention on Tobacco Control ratified parties | Indian Journal of Medical Research | No | P-R |
| **238** | Schwartz R, Chaiton M et al. | 2018 | Tobacco industry tactics in preparing for menthol ban | Tobacco Control | No | P-R |
| **239** | Scollo M, Bayly M et al. | 2018 | Tobacco product developments in the Australian market in the 4 years following plain packaging | Tobacco Control | No | P&L |
| **240** | Stead M, Eadie D et al. | 2018 | Tobacco companies' use of retailer incentives after a ban on point-of-sale tobacco displays in Scotland | Tobacco Control | No | TAPS |
| **241** | Astuti PAS, Kurniasari NMD et al. | 2019 | From glass boxes to social media engagement: an audit of tobacco retail marketing in Indonesia | Tobacco Control | No | TAPS |
| **242** | Baker J & Diethelm P | 2019 | World: PMI's continued subliminal promotion of combustible products in F1 motor sports | Tobacco Control | Yes | TAPS |
| **243** | Borland T, D'Souza SA, et al. | 2019 | Is blue the new green? Repackaging menthol cigarettes in response to a flavour ban in Ontario, Canada | Tobacco Control | No | P-R |
| **244** | Evans-Reeves KA, Hiscock R et al. | 2019 | Prospective longitudinal study of tobacco company adaptation to standardised packaging in the UK: identifying circumventions and closing loopholes | BMJ Open | No | P&L, P-R |
| **245** | Iacobelli M, Saraf S et al. | 2019 | Manipulated: graphic health warnings on smokeless tobacco in rural India | Tobacco Control | No | P&L |
| **246** | Jackler RK, Li VY, et al. | 2019 | Promotion of tobacco products on Facebook: policy versus practice | Tobacco Control | No | TAPS |
| **247** | Lindblom EN, Johnson AC et al. | 2019 | How and why consumers view "Little Cigars" as legally defined cigarettes | Tobacco Regulatory Science, 5(2) | No | P-R |
| **248** | Moodie C, Thrasher JF et al. | 2019 | Flavour capsule cigarettes continue to experience strong global growth. | Tobacco Control | No | P-R |

**Supplementary material**

Table 1: List of included sources

| | | | | | | |
|---|---|---|---|---|---|---|
| **249** | Purves RI, Moodie C et al. | 2019 | The Response of Retailers in Scotland to the Standardised Packaging of Tobacco Products Regulations and Tobacco Products Directive | Nicotine & Tobacco Research | No | P&L |
| **250** | Reynales-Shigematsu L, Wipfli H et al. | 2019 | Tobacco control in Mexico: a decade of progress and challenges | Salud Pública de México | No | P&L, SF, TAPS |
| **251** | Tanigaki J & Poudyal H | 2019 | Challenges and opportunities for greater tobacco control in Japan | International Journal of Drug Policy | No | SF |
| **252** | van der Eijk Y, Lee JK & Ling PM | 2019 | How Menthol Is Key to the Tobacco Industry's Strategy of Recruiting and Retaining Young Smokers in Singapore | Journal of Adolescent Health | No | TAPS |
| **253** | Yadav A, Ling P & Glantz S | 2019 | Smokeless tobacco industry's brand stretching in India | Tobacco Control | No | TAPS |
| **254** | Amul GGH, Tan GPP & van der Eijk Y | 2020 | A Systematic Review of Tobacco Industry Tactics in Southeast Asia: Lessons for Other Low- And Middle-income Regions | International Journal of Health Policy and Management | No | P&L, TAPS |
| **255** | Astuti PAS, Assunta M & Freeman B | 2020 | Why is tobacco control progress in Indonesia stalled? - a qualitative analysis of interviews with tobacco control experts | BMC Public Health | No | TAPS |
| **256** | Baker J | 2020 | Southeast Asia: tobacco industry's 'smoke-free' duplicity | Tobacco Control | Yes | P&L, P-R |
| **257** | Baker J | 2020 | World: PMI in global motorsport smoke-free PR spin | Tobacco Control | Yes | TAPS |
| **258** | Baker J | 2020 | Europe/UK: menthol cigarettes & flavoured tobacco ban | Tobacco Control | Yes | P-R |
| **259** | Bhatta DN, Bialous S et al. | 2020 | Exceeding WHO Framework Convention on Tobacco Control (FCTC) Obligations: Nepal Overcoming Tobacco Industry Interference to Enact a Comprehensive Tobacco Control Policy | Nicotine & Tobacco Research | No | TAPS |
| **260** | Bhatta DN, Crosbie E et al. | 2020 | Defending Comprehensive Tobacco Control Policy Implementation in Nepal from Tobacco Industry Interference (2011–2018) | Nicotine & Tobacco Research | No | P&L, SF, TAPS |
| **261** | Cho, SM, Saw YM et al. | 2020 | Cross-sectional study on tobacco advertising, promotion and sponsorship (TAPS) and violations of tobacco sale regulations in Myanmar: do these factors affect current tobacco use among Myanmar high school students? | BMJ Open | No | P&L, TAPS |
| **262** | Dewhirst T & Lee WB | 2020 | Kent cigarette brand marketing in the Republic of Korea: the role of a pioneering image, flavour capsules and leader price promotions | Tobacco Control | No | P&L |
| **263** | Hefler M & Baker J | 2020 | UK: Tobacco companies investigated over menthol ban | Tobacco Control | Yes | P-R |
| **264** | Hiscock R, Silver K et al. | 2020 | Tobacco industry tactics to circumvent and undermine the menthol cigarette ban in the UK | Tobacco Control | No | P-R, TAPS |
| **265** | Lindblom EN, Mays D et al. | 2020 | Has FDA abandoned its efforts to make fake-cigar cigarettes comply with federal tobacco control laws that apply to cigarettes but not cigars? | Tobacco Control | No | P-R |

**Supplementary material**

Table 1: List of included sources

| | | | | | | |
|---|---|---|---|---|---|---|
| **266** | Watts C, Burton S et al. | 2020 | 'Friends with benefits': how tobacco companies influence sales through the provision of incentives and benefits to retailer | Tobacco Control | No | TAPS |
| **267** | Weiger CV, Smith K et al. | 2020 | Cigarette Packs With URLs Leading to Tobacco Company Websites: Content Analysis | Journal of Medical Internet Research | No | TAPS |
| **268** | Arnold D | 2021 | The Netherlands: Retailers caught breaching tobacco display & advertising bans | Tobacco Control | Yes | TAPS |
| **269** | Azzahro EA, Dewi DMSK et al. | 2021 | Two tobacco retailer programmes in Banyuwangi, Indonesia: a qualitative study | Tobacco Control | No | TAPS |
| **270** | Branston JR, Hiscock R et al. | 2021 | Cigarette-like cigarillo introduced to bypass taxation, standardised packaging, minimum pack sizes, and menthol ban in the UK | Tobacco Control | No | P&L, P-R |
| **271** | Chaiton MO, Schwartz R et al. | 2021 | The use of flavour cards and other additives after a menthol ban in Canada | Tobacco Control | No | P-R |
| **272** | Chandra S, Rinkoo AV et al. | 2021 | Tobacco Advertising, Promotion and Sponsorship in India and Indonesia: Present Regime and the Way Forward | Asian Pacific Journal of Cancer Prevention | No | P-R, TAPS |
| **273** | Cohen JE, Welding K et al. | 2021 | The Flavor Train: The Nature and Extent of Flavored Cigarettes in Low- and Middle-Income Countries | Nicotine & Tobacco Research | No | P-R |
| **274** | Crosbie E, Defrank V et al. | 2021 | Tobacco supply and demand strategies used in African countries | Bulletin of the World Health Organization | No | P&L, TAPS |
| **275** | Hefler M, Bianco E et al. | 2021 | What facilitates policy audacity in tobacco control? An analysis of approaches and supportive factors for innovation in seven countries | Tobacco Control | No | P&L, TAPS |
| **276** | Ichikawa M, Hori A et al. | 2021 | Intensified advertising of heated tobacco products in Japan: an apparent shift in marketing strategy | Tobacco Control | No | TAPS |
| **277** | Kong J, Chu S et al. | 2021 | The tobacco industry and electronic cigarette manufacturers enjoy a loophole in the legal definition of tobacco in South Korean law | Tobacco Control | No | TAPS |
| **278** | Naznin E, Wynne O et al. | 2021 | Smokeless tobacco policy in Bangladesh: A stakeholder study of compatibility with the World Health Organization's Framework Convention on Tobacco Control | Drug and Alcohol Review | No | TAPS |
| **279** | Polanska K & Kaleta D | 2021 | Tobacco and e-cigarettes point of sale advertising – assessing compliance with tobacco advertising, promotion and sponsorship bans in Poland | International Journal of Environmental Research and Public Health | No | TAPS |
| **280** | Stubbs T | 2021 | Commercial determinants of youth smoking in ASEAN countries: A narrative review of research investigating the influence of tobacco advertising, promotion, and sponsorship | Tobacco Induced Diseases | No | TAPS |
| **281** | Udokanma EE, Ogamba I & Ilo C | 2021 | A health policy analysis of the implementation of the National Tobacco Control Act in Nigeria | Health Policy and Planning | No | P&L |
| **282** | Watts C, Burton S & Freeman B | 2021 | 'The last line of marketing': covert tobacco marketing tactics as revealed by former tobacco industry employees | Global Public Health | No | TAPS |
| **283** | Welker M | 2021 | Indonesia's Cigarette Culture Wars: Contesting Tobacco Regulations in the Postcolony | Comparative Studies in Society and History | No | P&L |

**Supplementary material**

Table 1: List of included sources

| | | | | | | |
|---|---|---|---|---|---|---|
| **284** | Yadav A & Glantz, SA | 2021 | Tobacco imagery in entertainment media: Evolution of tobacco-free movies and television programmes rules in India | BMJ Global Health | No | TAPS |
| **285** | Yi J, Kim J & Lee S | 2021 | British American Tobacco's 'Glo Sens' promotion with K-pop | Tobacco Control | No | TAPS |
| **286** | Brink AL, Glahn AS & Kjaer NT | 2022 | Tobacco companies' exploitation of loopholes in the EU ban on menthol cigarettes: a case study from Denmark | Tobacco Control | No | P-R |
| **287** | Crespi E, Iacobelli M et al. | 2022 | Industry manipulation of pictorial health warning labels in Pakistan | Tobacco Control | No | P&L |
| **288** | Crosbie E, Gutkowski P et al. | 2022 | Progress in adopting bans on tobacco advertising, promotion, and sponsorship in the Americas: lessons from Uruguay and Argentina | Revista Panamericana de Salud Pública | No | TAPS |
| **289** | Cunningham R | 2022 | Canada implements "Slide and shell" packaging for cigarettes | Tobacco Control | Yes | P&L |
| **290** | Evans-Reeves K, Lauber K & Hiscock R | 2022 | The 'filter fraud' persists: the tobacco industry is still using filters to suggest lower health risks while destroying the environment | Tobacco Control | No | P&L, P-R, TAPS |
| **291** | Ghose S, Mahajan A & Datta SS | 2022 | A public policy analysis with key stakeholders' insights to understand India's compliance with the WHO Framework Convention on Tobacco Control | ecancermedicalscience | No | TAPS |
| **292** | Ling PM, Kim M et al. | 2022 | Moving targets: how the rapidly changing tobacco and nicotine landscape creates advertising and promotion policy challenges | Tobacco Control | No | P-R |
| **293** | Stubbs T, White V et al. | 2022 | Tobacco industry influence in low- and middle-income countries in the ASEAN region: Qualitative interviews with tobacco control experts during the COVID-19 pandemic | Tobacco Induced Diseases | No | TAPS |
| **294** | van der Eijk Y & Yang AY | 2022 | Tobacco industry marketing adaptations to Singapore plain packaging | Tobacco Control | No | P&L |
| **295** | Briggs J & Seidenberg AB | 2023 | USA: tobacco companies target customers with menthol alternatives to circumvent upcoming California sales ban | Tobacco Control | Yes | P-R |
| **296** | Evans-Reeves K | 2023 | Africa Goodbye, PMI: African Tobacco Control Alliance ensures Philip Morris International | Tobacco Control | Yes | TAPS |
| **297** | Kapoor S & Yadav A | 2023 | India: PMI yet again circumvents Indian laws, this time to promote banned electronic cigarettes and other nicotine and tobacco devices | Tobacco Control | Yes | P-R |
| **298** | Kyriakos CN, Chung-Hall J et al. | 2023 | Optimising a product standard for banning menthol and other flavours in tobacco products | Tobacco Control | No | P-R |
| **299** | Meza LR, Galimov A et al. | 2023 | Proliferation of 'non-menthol' cigarettes amid a state-wide flavour ban | Tobacco Control | No | P-R |
| **300** | Page MK, Paul EE et al. | 2023 | Still 'Cool': tobacco industry responds to state-wide menthol ban with synthetic coolants | Tobacco Control | No | P-R |

**Supplementary material**

Table 1: List of included sources

| | | | | | | |
|---|---|---|---|---|---|---|
| **301** | Perera PADS, Gnanarathna DJA et al. | 2023 | Sri Lanka: tobacco industry pushes back against flavour ban | Tobacco Control | Yes | P-R |
| **302** | Stubbs T, White VM et al. | 2023 | Uncovering the advertising and promotional strategies of tobacco companies in Cambodia: recall of below-the-line marketing among young male smokers | Tobacco Control | No | TAPS |
| **303** | Suarjana K, Mulyawan KH et al. | 2023 | Factors Associated with The Compliance of Tobacco Advertisement, Promotion and Sponsorship (TAPS) Ban in Bali, Indonesia | Malaysian Journal of Medicine and Health Sciences | No | TAPS |
| **304** | Tselengidis A, Adams S et al. | 2023 | From the international tobacco control arena to the local context: a qualitative study on the tobacco advertising, promotion and sponsorship legislative environment in Sudan and the challenges characterising it | BMJ Open | No | TAPS |
| **305** | Tselengidis A, Adams S et al. | 2023 | Achieving a tobacco-free Bangladesh by2040: a qualitative analysis of the tobacco advertising environment and prohibitions in Bangladesh | BMJ Open | No | TAPS |
| **306** | Abdullah SM, Huque R et al. | 2024 | Non-compliant packaging and illicit smokeless tobacco in Bangladesh, India and Pakistan: findings of a pack analysis | Tobacco Control | No | P&L, P-R |
| **307** | Alaouie H & Nakkash R | 2024 | Lebanon: Tobacco industry ignores marketing and advertising regulations | Tobacco Control | Yes | TAPS |
| **308** | Kyriakos CN, Filippidis F et al. | 2024 | Use of Menthol Cigarettes and Accessories Among Youth Who Smoked After the Menthol Cigarette Ban in England and Canada, 2021: Implications for Health Equity | Nicotine & Tobacco Research | No | P-R |

*P&L - Packaging and Labelling, P-R - Product-related, SF - Smoke-free, TAPS - Tobacco Advertising, Promotion and Sponsorship
